# Broadly neutralizing antibodies from an individual that naturally cleared multiple hepatitis C virus infections uncover molecular determinants for E2 targeting and vaccine design

**DOI:** 10.1371/journal.ppat.1007772

**Published:** 2019-05-17

**Authors:** Zhen-Yong Keck, Brian G. Pierce, Patrick Lau, Janine Lu, Yong Wang, Alexander Underwood, Rowena A. Bull, Jannick Prentoe, Rodrigo Velázquez-Moctezuma, Melanie R. Walker, Fabio Luciani, Johnathan D. Guest, Catherine Fauvelle, Thomas F. Baumert, Jens Bukh, Andrew R. Lloyd, Steven K. H. Foung

**Affiliations:** 1 Department of Pathology, Stanford University School of Medicine, Stanford, California, United States of America; 2 University of Maryland Institute for Bioscience and Biotechnology Research, Rockville, Maryland, United States of America; 3 Department of Cell Biology and Molecular Genetics, University of Maryland, College Park, Maryland, United States of America; 4 Viral Immunology Systems Program, The Kirby Institute and School of Medical Sciences, University of New South Wales, Sydney, NSW, Australia; 5 Copenhagen Hepatitis C Program (CO-HEP), Department of Infectious Diseases, Hvidovre Hospital and Department of Immunology and Microbiology, Faculty of Health and Medical Sciences, University of Copenhagen, Copenhagen, Denmark; 6 Inserm U1110, Institut de Recherche sur les Maladies et Hépatiques, Strasbourg, France; 7 Université de Strasbourg, Strasbourg, France; 8 Pole Hépato-digestif, Institut Hospitalo-Universitaire, Hopitaux Universitaires de Strasbourg, Strasbourg, France; Institut Pasteur, FRANCE

## Abstract

Cumulative evidence supports a role for neutralizing antibodies contributing to spontaneous viral clearance during acute hepatitis C virus (HCV) infection. Information on the timing and specificity of the B cell response associated with clearance is crucial to inform vaccine design. From an individual who cleared three sequential HCV infections with genotypes 1b, 1a and 3a strains, respectively, we employed peripheral B cells to isolate and characterize neutralizing human monoclonal antibodies (HMAbs) to HCV after the genotype 1 infections. The majority of isolated antibodies, designated as HMAbs 212, target conformational epitopes on the envelope glycoprotein E2 and bound broadly to genotype 1–6 E1E2 proteins. Further, some of these antibodies showed neutralization potential against cultured genotype 1–6 viruses. Competition studies with defined broadly neutralizing HCV HMAbs to epitopes in distinct clusters, designated antigenic domains B, C, D and E, revealed that the selected HMAbs compete with B, C and D HMAbs, previously isolated from subjects with chronic HCV infections. Epitope mapping studies revealed domain B and C specificity of these HMAbs 212. Sequential serum samples from the studied subject inhibited the binding of HMAbs 212 to autologous E2 and blocked a representative domain D HMAb. The specificity of this antibody response appears similar to that observed during chronic infection, suggesting that the timing and affinity maturation of the antibody response are the critical determinants in successful and repeated viral clearance. While additional studies should be performed for individuals with clearance or persistence of HCV, our results define epitope determinants for antibody E2 targeting with important implications for the development of a B cell vaccine.

## Author summary

Studies of hepatitis C virus (HCV) infected individuals spontaneously clearing acute infections provide an opportunity to characterize the specificities of associated protective antibody responses. In an individual who resolved three separate HCV infections with different HCV genotypes, the antibodies induced during these acute infection episodes were similar to those induced during chronic infection. Surprisingly, the earliest detected antibodies were directed against conformational HCV epitopes on the envelope glycoprotein E2 (including polyprotein residues 434–446) known to be targeted by broadly neutralizing antibodies. Taken together, the key B-cell determinants in spontaneous clearance are the timing and affinity maturation of broadly neutralizing antibody responses.

## Introduction

Over 70 million people worldwide are infected with hepatitis C virus (HCV), with an annual mortality of approximately 400,000 associated with liver failure and hepatocellular carcinoma [1, 2]. Encouragingly for patients with chronic infections with HCV, advances in understanding of HCV virology have led to the development of virus-specific direct acting antivirals (DAAs) [3, 4]. However, the large majority of infected patients remain undiagnosed and/or live in countries with limited resources and with minimal or no access to DAA-based therapies. Indeed, global access to DAAs has been estimated to be less than 10% of HCV infected individuals [5]. Thus, the prevention of global spread and eradication of HCV infection will require a protective vaccine. A necessary step in the design of an effective vaccine is to identify relevant mechanisms of immune protection. Vigorous and sustained CD4+ and CD8+ T cell responses are associated with successful clearance, which occurs in 25% of acute infection episodes [6]. While humoral immunity has been traditionally thought to have a minor role in controlling acute HCV infection, emerging evidence supports the importance of neutralizing antibodies in spontaneous viral clearance. The induction of a neutralizing antibody response in the acute phase of infection has been associated with clearance of infection in single source outbreaks of acute HCV infection [7]. Further, control of acute infection has been associated with the early appearance of neutralizing antibody responses [8]. Broad reactivity of these neutralizing antibody responses appears to be associated with viral clearance. However, further information is needed on the timing and specificity of the neutralizing antibody responses associated with viral clearance during acute infection and reinfection, and better understanding on how these responses differ from those found during chronic infection will inform vaccine design.

We have addressed these issues by characterizing a panel of human monoclonal antibodies (HMAbs) isolated from peripheral B cells of an individual that sequentially cleared genotypes 1b and 1a HCV infections, as well as subsequently clearing a genotype 3a HCV infection. Neutralizing HMAbs from B cells obtained after resolved infections with genotype 1b and 1a HCV strains demonstrated broad reactivity and targeted overlapping conformational epitopes in highly immunogenic clusters that are similar to those that have been characterized from HCV infected subjects during chronic infections. Interestingly, the earliest neutralizing antibodies induced in this individual were directed against a region on the E2 glycoprotein (including residues 434–446) that is likely to be of low immunogenicity but is highly conserved. The early appearance of these antibodies suggests an important role in viral clearance. Overall, these findings support that natural clearance of acute HCV infection is associated with broadly neutralizing antibodies that are similar to those observed during chronic infection, and that key determinants in spontaneous clearance are the timing and affinity maturation of this response.

## Results

### Studied subject

We studied an individual, designated as 300212, who had three documented episodes of acute HCV infection acquired through injection drug use over a course of 320 weeks. Each episode was associated with spontaneous clearance. Sequential samples of blood were obtained to isolate peripheral blood mononuclear cells (PBMCs) and serum. The subject was a 21-year-old, immunocompetent male who screened negative for HIV, HBV and HCV antibodies before enrollment in an ongoing prospective cohort study of high risk, uninfected injection drug users—the Hepatitis C Incidence and Transmission Study in prisons (HITS-p). He was HCV seropositive and RNA positive with a genotype 1b infection at 41 weeks after his initial evaluation, and 21 weeks after his first estimated infection (midpoint between his last known HCV antibody negative test and first antibody positive test) (S1 Table) [9]. The collection dates described in this report are designated as the number of weeks after this first estimated date of infection. He was also found to have the *IL28B* rs12979860 polymorphism associated with spontaneous viral clearance [10]. On enrollment into HITS-p, he reported that he started injecting drugs four years earlier. During regular follow-up in HITS-p, he reported periods of daily injecting drug use and sharing of needles with other inmates. At week 76 post-infection testing, he was HCV RNA negative, but remained HCV seropositive. At week 122, he became transiently HCV RNA positive with a second infection, with a genotype 1a isolate. After only one week of viremia, he became HCV RNA negative. This aviremic status persisted until week 277, when he was diagnosed with a third infection with a genotype 3a isolate. Viremia continued until week 303, but was not detected at week 320.

### A broadly neutralizing antibody response was associated with viral clearance

**Fig 1:**
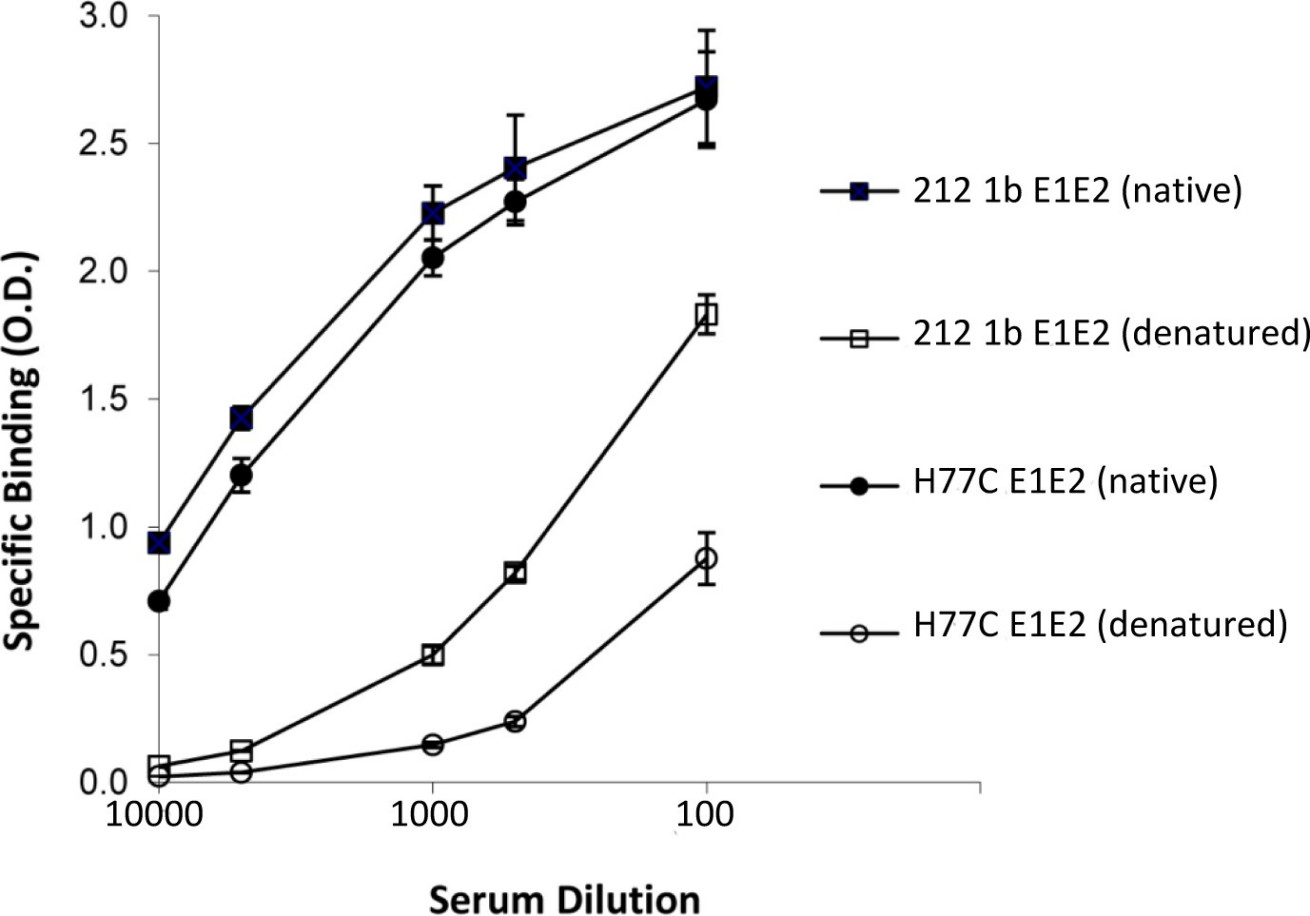
Serum antibodies from individual 300212 with acute resolving HCV infections are mainly to conformational virus envelope determinants. Recombinant autologous 212 1b (square symbol) and H77C (circle symbol) E1E2 lysates were either left untreated (native, solid symbols) or denatured (open symbols). After treatment, the proteins were captured by pre-coated GNA wells. Bound proteins were incubated with week 76 serum diluted at 1:100, 500, 1000, 5000, and 10,000 (*x*-axis). Bound antibodies were detected as described in Materials and Methods. The *y*-axis shows the mean optical density values for triplicate wells, the mean of two experiments ±SD.

To assess whether neutralizing antibodies contributed to viral clearance of the first two episodes of acute HCV infection in the individual 300212, serum serial dilutions, 1:100, 1:500, 1:1000, 1:5000 and 1:10,000, at each timepoint from week 21 to 182 (except for week 161) were tested for binding and neutralizing activities against the autologous 1b isolate and a heterologous 2a isolate (Table 1). Thus, an autologous genotype 1b HCVpp was constructed from the first infection at week 21, termed 212 1b HCVpp. For the second and third infections, recovery of autologous E1E2 was not successful. Serum antibody binding was determined against cell lysates expressing 212 1b recombinant E1E2 by ELISA. To begin to assess breadth of neutralization, one other isolate was tested, JFH1 2a HCVcc. In the viremic phase of the first infection at week 21, significant binding for serum antibodies to autologous E1E2 was only detected at 1:100 dilution (as defined by >0.5 optical density (O.D.)). However, no significant neutralizing activities were present (defined by ≥50% neutralization, IC_50_) at this timepoint even at a reciprocal serum dilution of 100 (Table 1). By contrast at week 76, when the subject was not viremic, peak antibody binding titer of 5000 was observed against 212 1b E1E2. Neutralizing serum antibody titers of 500 and 1000 were observed, respectively, against autologous 212 1b HCVpp and heterologous 2a HCVcc. The presence of neutralizing activity against a heterologous isolate is consistent with the induction of broadly reactive neutralizing antibodies. While it is not known when in the weeks 21 and 76 interval that clearance occurred, a pattern of increasing binding and neutralizing antibody titers was observed. To assess whether this response at week 76 was directed against conformational epitopes on E1E2, serum antibody binding studies were performed employing native and denatured antigens. As shown by significant reduction in antibody binding titers to denatured autologous 212 1b and heterologous H77C 1a E1E2 antigens (Fig 1), the antibody responses were directed mainly at conformational epitopes. The subject did not have further follow-up until week 122, when a second infection with a genotype 1a isolate was detected at a lower viral load than the first infection (Table 1). Serum antibody binding titer at week 122 was reduced to 1000 against 212 1b, but neutralization was maintained at 500 against this isolate. Seven days later at week 123, spontaneous viral clearance had occurred. An increase in neutralizing titer to 1000 was observed at week 135 against 212 1b HCVpp. At weeks 150 and 182, neutralizing titers were 100 and 500 against 212 1b HCVpp and 2a HCVcc, respectively.

**Table 1:** Serum antibody reactivity and neutralization against autologous HCVpp and neutralization against a heterologous HCVcc.

Timepoint	Genotype	Specific Binding*	Neutralization	
	Viral load (IU/ml)	212 1b*	212 1b HCVpp	2a HCVcc
21	1b 41802	100*	<100**	<100
76	-	5000	500	1000
122	1a 746	1000	500	500
123	-	1000	500	500
135	-	1000	1000	500
150	-	1000	100	500
182	-	1000	100	500

*Serum binding titer in which relative optical density is >0.5 to recombinant E1E2 by ELISA. Tested in triplicates and the mean of two experiments.

**Serum neutralization titer in which 50% inhibition (IC_50_) is calculated from dose-dependent neutralization. Tested twice in triplicates; representative experiment shown.

**Fig 2:**
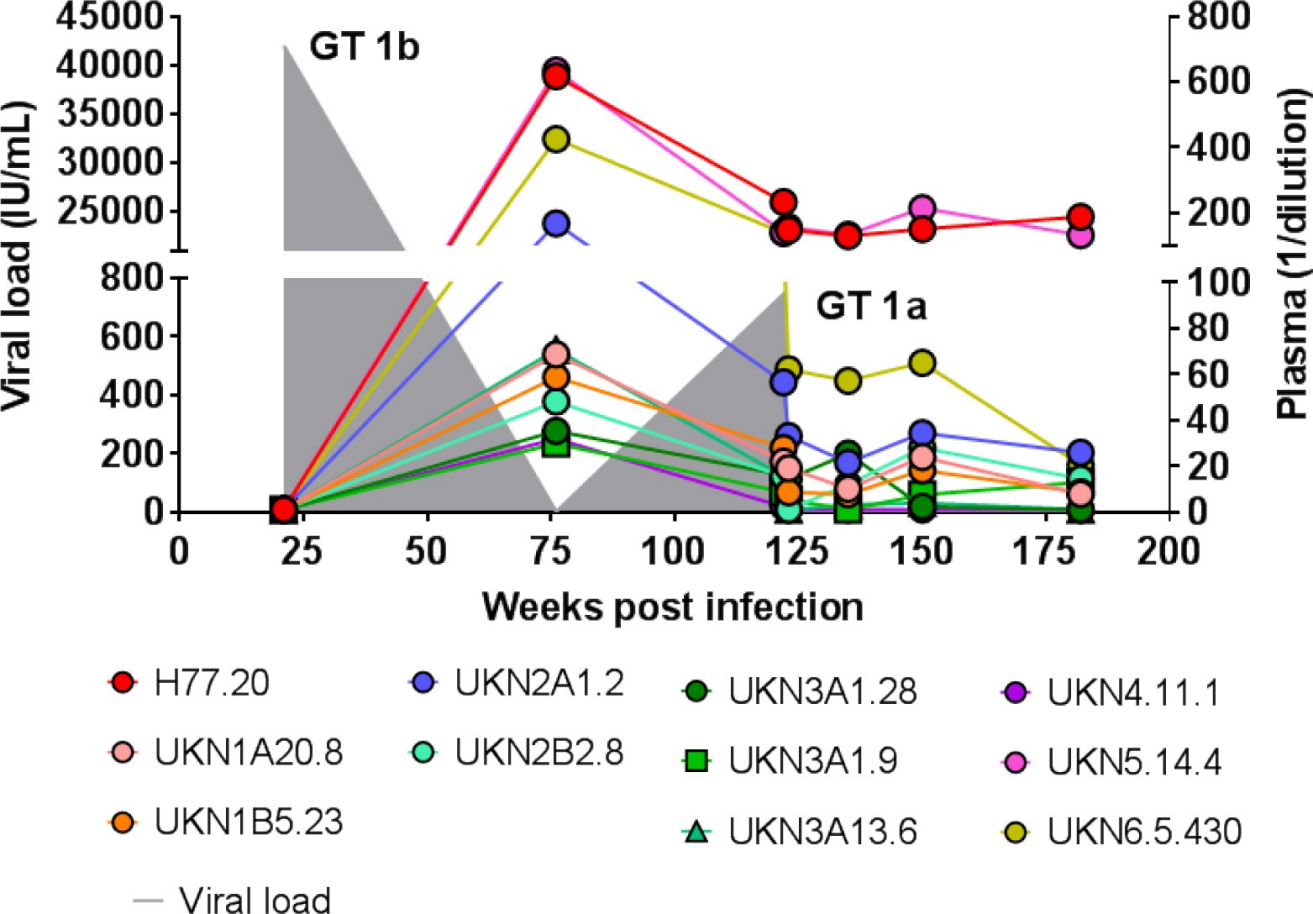
Serum IC_50_ neutralization titers at different timepoints against heterologous genotype HCVpp in individual 300212 with acute resolving HCV infections. Serum neutralization titers in which ≥ 50% neutralization was observed (IC_50_) against the 11 tested HCVpp were calculated using a non-linear regression analysis (Log inhibitor vs normalized response) in GraphPad PRISM V7.02 (see S1–S4 Figs) and plotted longitudinally at defined time points *(x-axis*). The viral load with the stated genotype isolates at these defined time points is plotted as the grey shading (left *y-axis*).

To further assess the breadth of neutralizing antibody responses, serum neutralization at the same dilutions (with the addition of 1:50) from week 21 to 182 were tested against a panel of 11 heterologous HCVpp (Fig 2 and S1–S4 Figs). Consistent with the findings against autologous 212 1b HCVpp and a heterologous 2a HCVcc (Table 1), no significant neutralizing activity (defined by ≥50% neutralization) against any heterologous HCVpp was observed for week 21, even at a reciprocal serum dilution of 50 (S1–S4 Figs). By week 76, all heterologous genotype 1 HCVpp (S1 Fig) were neutralized with a minimum neutralizing titer of 50 and similarly, one of two genotype 2 HCVpp (S2 Fig), one of three genotype 3 HCVpp (S3 Fig), a single genotype 5 and a single genotype 6 HCVpp (S4 Fig). The variants, e.g., UKN3A1.9 and UKN4.11.1 (S3 and S4 Figs), that are poorly neutralized are likely escape/resistant isolates. Taken together, these findings at week 76 provide further evidence for the induction of broadly neutralizing antibodies (Fig 2). During the second infection (week 122), while cross-genotype neutralizing titers had dropped, neutralizing titers against other genotype 1 isolates remained at 50 or higher (Fig 2). Serum neutralizing titers after this timepoint decreased, except for the two isolates H77.20 (1a) and UKN5.14.4 (5a), for which they remained unchanged even after spontaneous viral clearance. Collectively, a pattern of no or low neutralizing activity at week 21 (initial viremia) that increased to higher neutralizing activity levels at week 76 against a genotype-diverse panel of HCVpp is indicative of broadly neutralizing antibodies being induced as part of the antibody response to the first infection. The second infection also was associated with development of higher neutralizing titers against an autologous isolate (from the first infection) temporally associated with viral clearance, observed at week 135 (Table 1).

### Human monoclonal antibodies to HCV isolated during viral clearance

To characterize the specificity of the antibody response, week 123 was selected for investigation. This timepoint was one week after the detection of the second HCV infection with a genotype 1a isolate at week 122, when viral RNA was no longer detected (Table 1). B cells isolated from PBMCs were used to construct a yeast scFv displayed library [11]. Of 600 monoclonal scFv cells that bound to E2, 27 having unique combinations of heavy and light chain CDR1, 2 and 3 regions were identified and converted to full IgG_1_ molecules. Of note are that two scFv clones, 212.1.1 and 212.9, accounted for more than 50% of the total clones analyzed. HMAbs 212.1.1 to 212.1.4 have the same VH but different VL. Similarly, 212.2.1 and 212.2.2, as well as 212.3.1 and 212.3.2, respectively have the same VH but different VL. Full-length IgG_1_ converted HMAbs were initially analyzed for their neutralization against heterologous H77C 1a HCVpp and JFH1 2a HCVcc, and autologous 212 1b HCVpp (Table 2). Fourteen of 27 E2 binding HMAbs neutralized at least one of two heterologous isolates, 1a H77C HCVpp and 2a JFH1 HCVcc, or autologous 212 HCVpp by >40%. The remaining 13 E2 binding HMAbs did not show significant neutralization activity. Among the 14 neutralizing antibodies, five HMAbs neutralized all three isolates, four HMAbs neutralized two isolates and five HMAbs neutralized one isolate. The last group of five antibodies neutralized only 2a JFH1 HCVcc, but not the autologous 212 HCVpp or heterologous 1a H77C HCVpp.

**Table 2:** Neutralization activity (%) of 212 human monoclonal antibodies.

	Antibody (20 μg/ml)																											
Isolates	212.1.1*	212.1.2	212.1.3	212.1.4	212.1.5	212.2.1	212.2.2	212.3.1	212.3.2	212.4	212.5	212.6	212.7	212.8	212.9	212.10	212.11	212.12	212.13	212.14	212.15	212.16	212.19	212.21	212.22	212.24	212.25	HC-11
1a H77C HCVpp	89	83	90	95	94	8	2	10	0	18	0	1	0	0	15	94	13	0	9	0	75	0	0	0	0	3	85	95
2a JFH1 HCVcc	80	87	51	34	24	27	60	71	14	60	69	67	30	36	89	92	13	9	15	23	43	20	2	6	20	28	70	94
212 HCVpp	74	58	66	69	61	25	7	11	5	33	31	33	35	35	87	61	14	18	33	30	20	11	6	6	12	2	58	58
0–40	* Selected antibodies (bold) for further studies.																											
40–100																												

### Broadly reactive HMAbs 212 are directed against conformational epitopes

**Fig 3:**
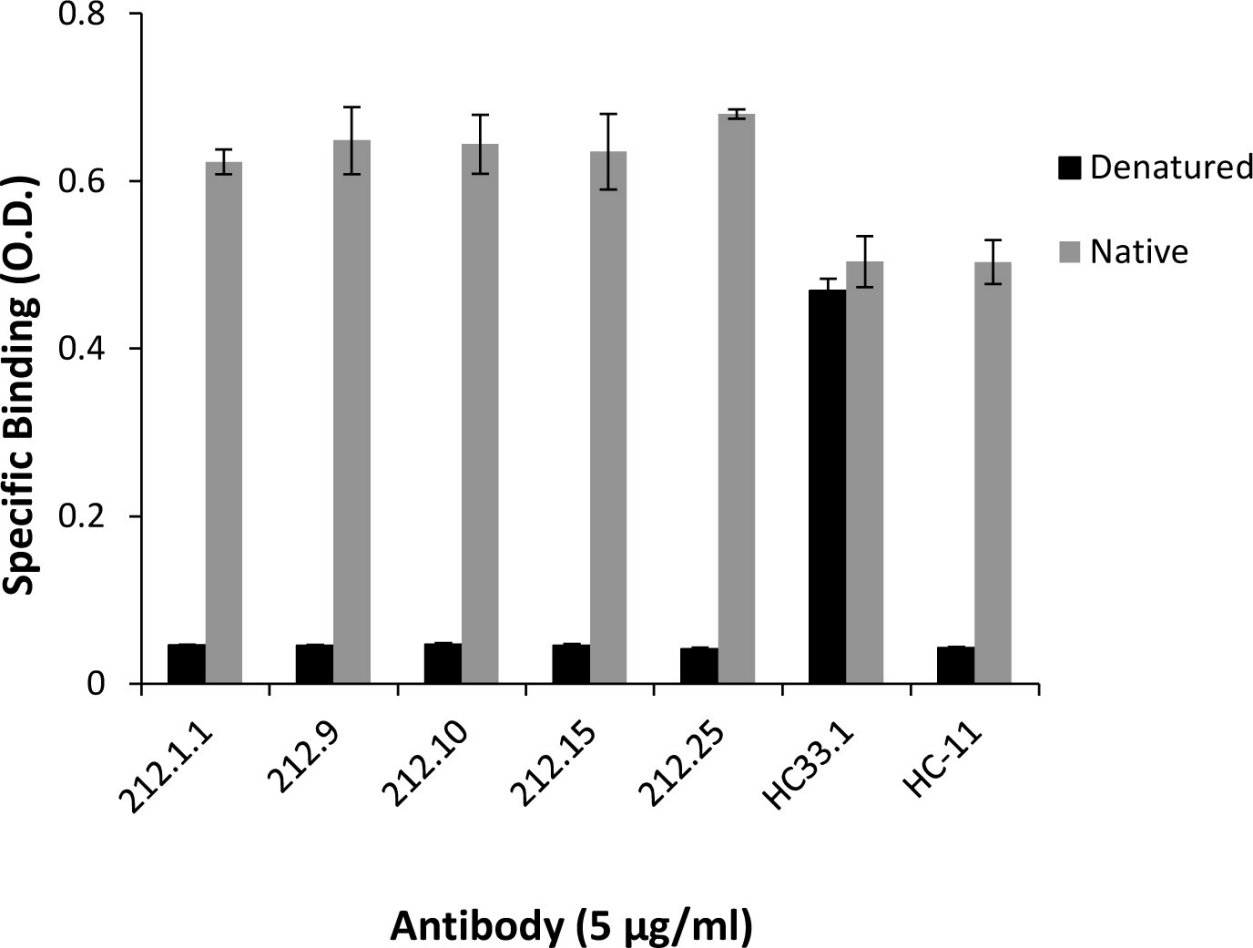
Selected HMAbs 212 do not bind to denatured autologous 212 1b HCV E2. Recombinant E1E2 lysate was either left untreated (native, gray bars) or denatured (black bars). After treatment, the proteins were captured by pre-coated GNA wells. Bound proteins were incubated with each 212 HMAb at 5 μg/ml (*x*-axis) and two control HMAbs, HC33.1 and HC-11 [12, 53]. Bound antibody was detected as described in Materials and Methods. The *y*-axis shows the mean optical density values for triplicate wells, the mean of two experiments ± SD.

Five HMAbs were selected for additional studies, 212.1.1, 212.9, 212.10, 212.15 and 212.25 that represent the range in neutralization patterns (i.e. against all three isolates, or two with or without the autologous isolate). Cross-reactivity of the HMAbs was examined against ten E1E2 proteins derived from six different HCV genotypes and subtypes (S2 Table). Broad binding patterns were observed for all antibodies, except for 212.1.1 that bound to only three of ten isolates. Two HMAbs, 212.10 and 212.25 bound to all ten isolates. Lower reactivity against genotype 3a was generally observed. Denaturation of 1b 212 E1E2 completely abrogated the binding reactivity for all five HMAbs by ELISA, demonstrating that these antibodies are directed against conformational epitopes on HCV E1E2 (Fig 3). As controls, HC33.1, an antibody directed to a predominantly linear epitope on the E2 glycoprotein, retained 80% binding [12] and HC-11, an antibody to a conformational epitope on the E2 glycoprotein, lost more than 95% binding to denatured E2 [13].

### Identification of the 212.10 HMAb as a cross genotype 1–6 neutralizing antibody

To further examine the cross-genotype neutralization potential of HMAbs 212, 212.1.1, 212.10 and 212.25 were tested in dose-response focus forming unit (FFU) reduction neutralization assays [14] against an HCVcc genotype panel consisting of genotypes 1a (strains H77 and TN), 1b (J4 and DH1), 2a (J6), 2b (J8), 3a (S52 and DBN), 4a (ED43), 5a (SA13) and 6a (HK6a) (Table 3) [4, 15–20]. No neutralization activity was observed for the negative control HMAb R04 (targeting HCMV) against any of these HCVcc, whereas the positive control antibody HC84.27 neutralized all tested HCVcc (S5–S8 Figs) [11]. Among the HMAbs 212, only 212.10 was cross-genotype reactive and neutralized all HCVcc strains tested except S52. Compared to HC84.27, 212.10 was more effective against DBN and SA13, and less effective against H77, S52 and ED43. 212.1.1 neutralized HCVcc of genotypes 1a, 4a and 6a, whereas 212.25 did not neutralize any of the tested HCVcc (S5–S8 Figs). We previously reported that lack of neutralization against HCVcc could involve E2 hypervariable region 1 (HVR1) related antibody protection [21–24]. We thus tested the HMAbs 212 against HCVcc H77 lacking HVR1 [25], and found potent neutralization for 212.1.1 and 212.10, and neutralization potential for 212.25 (Table 3; S5 Fig); 212.25 had 670–7700 fold lower efficiency against H77_ΔHVR1_ than 212.1.1, 212.10 and HC84.27.

**Table 3:** Neutralization activity of human monoclonal antibodies selected from individual 212 and controls against HCVcc of genotypes 1–6.

				Antibody		
HCVcc (Core-NS2)	genotype	R04	212.1.1	212.10	212.25	HC84.27
H77	1a	<15	**41 (36)**	**50 (15)**	<15	**97 (1.2)**
H77_ΔHVR1_	1a	<15	**100 (0.0051)**	**100 (0.0017)**	**85 (3.4)**	**100 (0.00044)**
TN	1a	<15	**53 (18)**	**84 (3.8)**	<15	**91 (1.7)**
DH1	1b	<15	<15	**48 (24)**	<15	**48 (26)**
J4	1b	<15	<15	**97 (0.90)**	<15	**100 (0.34)**
J6	2a	<15	<15	**25 (68)**	<15	**44 (45)**
J8	2b	<15	<15	**59 (2.5)**	<15	**72 (2.3)**
S52	3a	<15	<15	<15	<15	**54 (19)**
DBN	3a	<15	<15	**95 (0.0083)**	<15	**16 (57)**
ED43	4a	<15	**40 (33)**	**79 (3.6)**	<15	**100 (0.12)**
SA13	5a	<15	<15	**94 (0.81)**	<15	**25 (41)**
HK6a	6a	<15	**26 (40)**	**99 (0.012)**	<15	**100 (0.024)**

The indicated HCVcc genotype recombinants were subjected to dose-response neutralization with the indicated antibodies (S5–S8 Figs). Values represent % neutralization at 25 μg/ml. In the cases where the data permitted the interpolation of IC_50_ values, these are given in parenthesis (in μg/ml). If more than 15%, but less than 50%, neutralization was observed at 25 μg/ml, an additional neutralization was carried out at 75 μg/ml, permitting the interpolation of an IC_50_ value by combining the data.

### Competition with antigenic domain B to E antibodies

Broadly neutralizing HMAbs against HCV are predominantly directed against epitopes in E2 (reviewed in [26]) and epitope mapping and competition analysis has revealed that many of these neutralizing antibodies are directed at overlapping epitopes, which can be grouped into four distinct clusters, designated as antigenic domains B, C, D and E. Three of these clusters, B, C and D, contain conformational epitopes, and E contains mainly linear epitopes on E2 [11–13, 27]. It should be noted that domains B and D antibodies are distinguished by their respectively shared contact residues on E2. But some epitopes within domains B and D do overlap with shared contact residues in the 441–443 region forming a domain B-D supersite of conformational epitopes on the exposed surface of E2 [28]. The five 212 HMAbs initially selected for further analysis were therefore examined for their reactivity to these antigenic domains by competition studies with HMAbs HC-11 (domain B), CBH-7 (domain C), HC84.27 (domain D) and HC33.1 (domain E) against autologous 212 1b recombinant E1E2 (S3 Table). HMAbs 212.1.1 and 212.10 blocked domains B, C and D HMAbs by >60%; HMAb 212.9 blocked mainly domains B and D; and HMAbs 212.15 and 212.25 blocked antigenic domain C. There was no significant competition (defined as >40%) between these 212 HMAbs and HMAb HC33.1 (domain E). The findings place 212.15 and 212.25 epitopes within domain C, and the remaining three, 212.1.1, 212.9 and 212.10, in domains B and/or D.

### Neutralizing antibodies are present throughout the course of repeated clearance associated with multiple HCV infections

**Fig 4:**
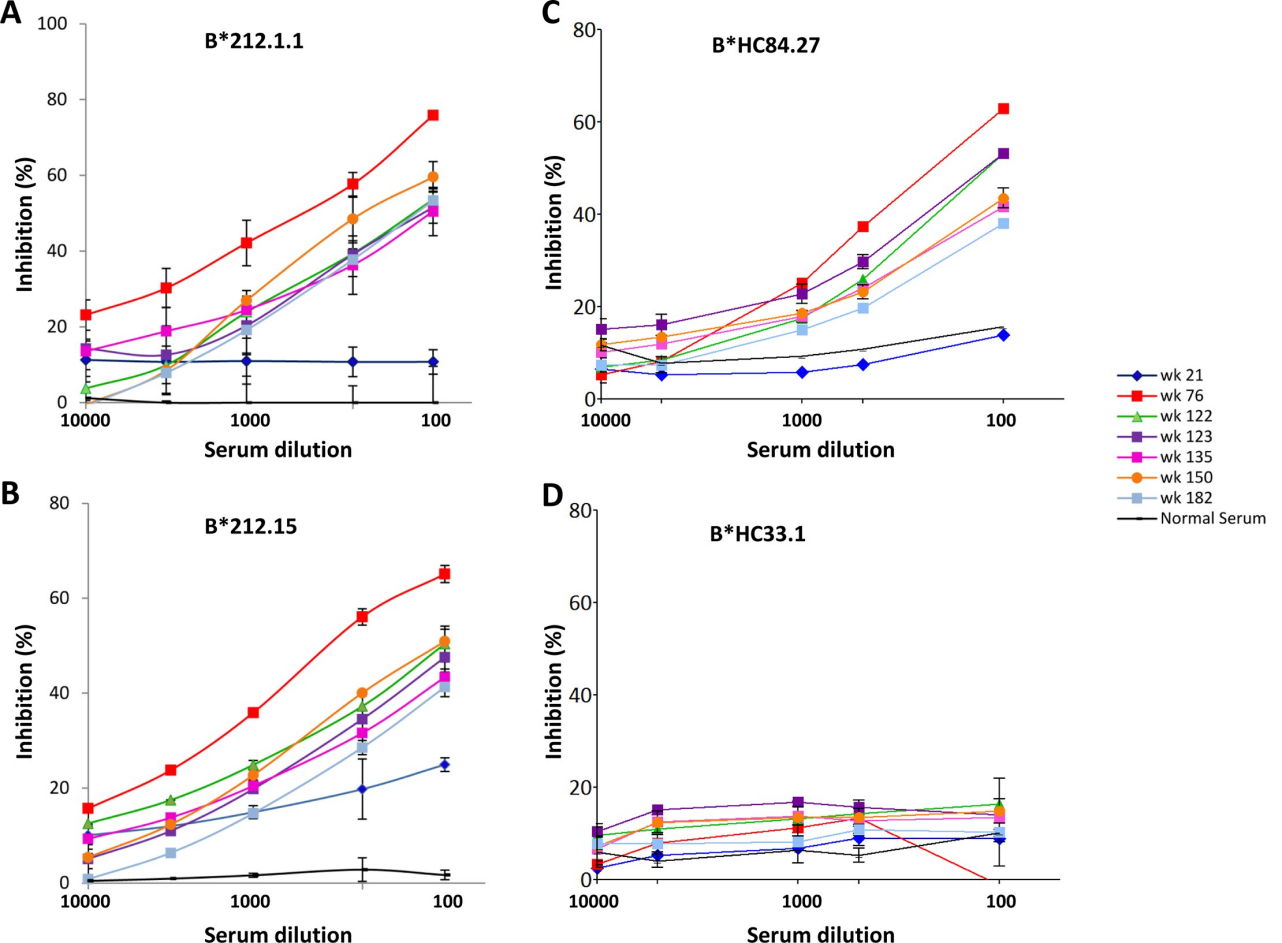
Sequential serum inhibition of HMAb 212 binding to autologous 212 1b E2 and domains D and E HMAbs to 1a H77C E2. Recombinant 212 1b E2 (A and B) or 1a H77C E2 (C and D) was captured by GNA in microtiter wells. The wells were then incubated with the indicated serum or normal serum control at dilutions of 100, 500, 1000, 5000 and 10000 for 30 minutes, followed by labeled HMAb 212.1.1 (A), 212.15 (B), HC84.27 (C) or HC33.1 (D) at 2 μg/ml. Binding was detected after anti-human IgG-labeled horseradish peroxidase. The *y*-axis shows the mean optical density values for triplicate wells, the mean of two experiments ±SD.

To address the question regarding whether the HMAbs isolated from this individual are part of a successful neutralizing antibody response, dilutions of sequential serum samples obtained throughout the course of the first two infections were tested for the presence of these HMAbs. Two antibodies, 212.1.1, and 212.15, were selected representing the antigenic domains B and C-like antibodies that have been isolated. Serum samples from week 21 to 182 (S1 Table) were diluted 1:100 to 1:10,000 and tested for their ability to block the binding of labeled 212.1.1 or 212.15 to recombinant 212 1b E1E2 (Fig 4A and 4B). No detectable inhibition was observed in association with the first genotype 1b infection at week 21. Dose-dependent inhibition was observed similarly with all subsequent serum samples, with some decrease against 212.15 HMAb at week 182. The patterns of domain B- and C-like antibodies being induced in response to the first two infections are consistent with their role in viral clearance. It is also possible that non-domain B- and C-like antibodies are induced that competed with 212.1.1 or 212.15.

### Global epitope mapping of 212 HMAbs

**Fig 5:**
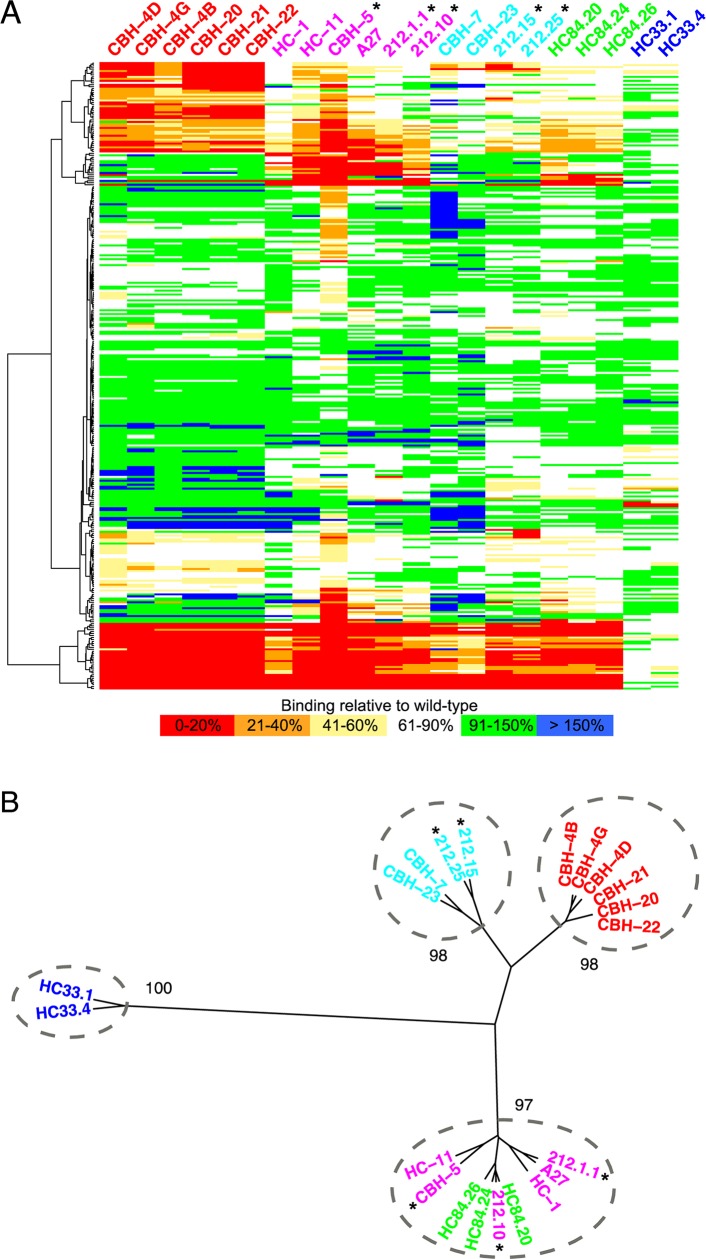
Global alanine scanning of E2 glycoprotein binding determinants. (A) Individual E2 alanine mutants were measured for binding affinity to four 212 HMAbs and HMAb CBH-5, which are shown along with 16 other HMAbs that were previously reported [29]. Affinities for each mutant are color coded based on percent binding with respect to wild-type E2, and residues (rows) were clustered using hierarchical clustering in R (www.r-project.org). Asterisks denote the HMAbs 212 and CBH-5, and antibody names are colored according to antigenic domains (A, red; B, magenta; C, cyan; D, green; E, blue). (B) Clustering of HMAbs based on global alanine scanning. Numbers show bootstrap confidence of clusters (out of 1.0), and clusters with bootstrap p-values greater than 0.95 are circled.

To definitively map the full set of E2 binding determinants of the HMAbs 212.1.1, 212.10, 212.15 and 212.25, we performed global alanine scanning of E2 with these four antibodies, in addition to CBH-5, which is a previously described antigenic domain B HMAb (Fig 5A) [27]. Epitope mapping of HMAb 212.9 was not performed because this antibody did not bind to or neutralize 1a H77C (S2 Table). After combining these measurements with results from our previously mapped panel of 16 HMAbs, which target five antigenic domains on E2 [29], we performed unsupervised clustering of this full set of antibodies (Fig 5B). This recapitulates initial assignment of HMAbs 212.15 and 212.25 as targeting antigenic domain C, while 212.1.1 and 212.10, in addition to CBH-5, are clustered with antibodies targeting the domain B-D supersite. HMAb 212.1.1 diverges from 212.10 based on global binding data and is clustered with antibodies targeting antigenic domain D, albeit with lower significance than the parent B-D supersite cluster (bootstrap probability 91%, versus 97% probability for the B-D supersite).

**Fig 6:**
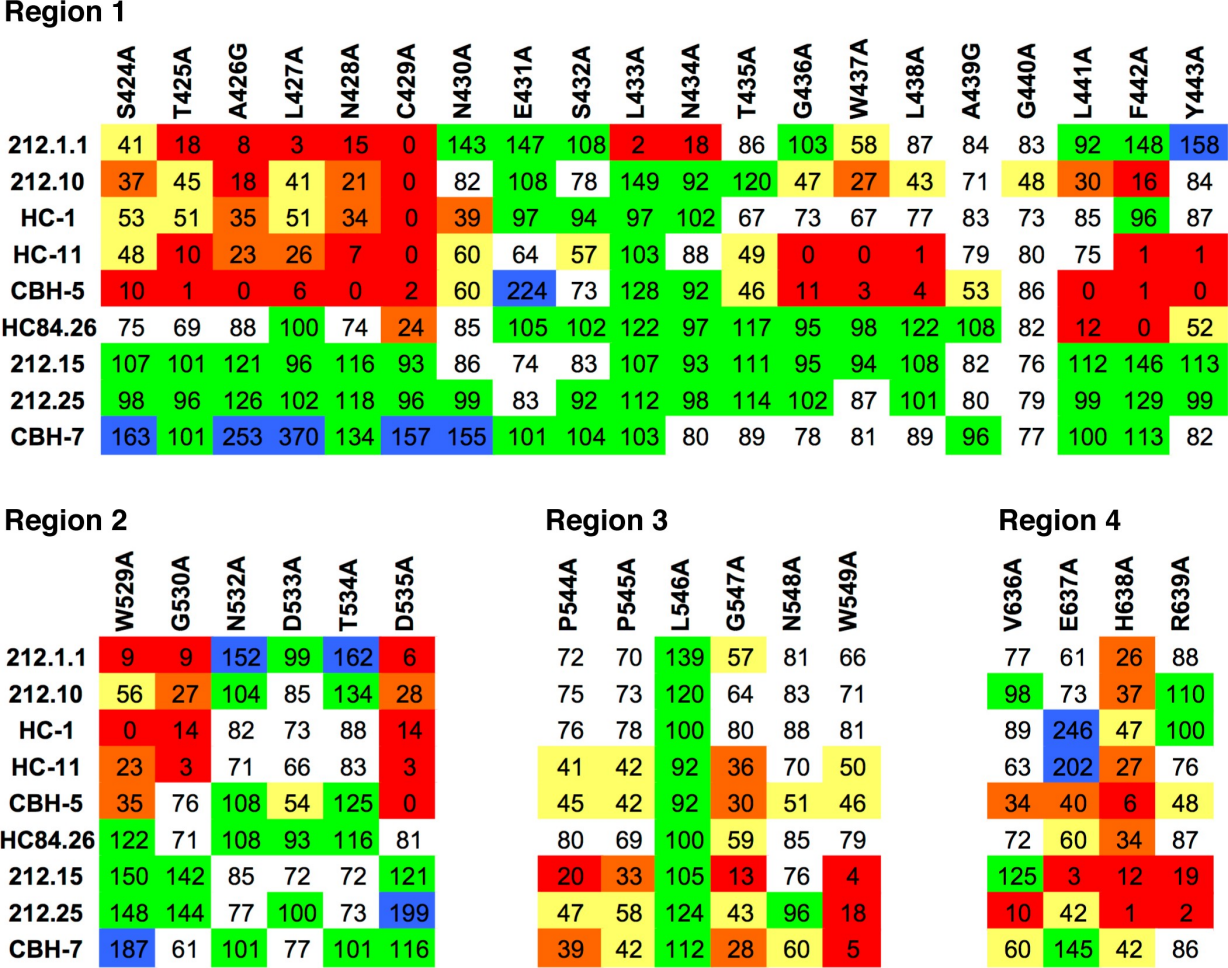
Epitope mapping for HCV E2. Epitope mapping data for HMAbs 212, as well as other HMAbs for reference, are shown for four regions of E2: Region 1 encompassing aa 424–443, Region 2, aa 529–535, Region 3, aa 544–549, and Region 4, aa 636–639. Cells show binding percentage relative to wt, and are color coded by percent binding as in Fig 5. Data are shown as mean values of two experiments performed in triplicate.

To highlight shared and differential residue-level effects on antibody recognition, the four 212 HMAbs are compared with CBH-5 and other HMAbs for four segments on E2 (respectively designated as regions 1, 2, 3, and 4 in Fig 6), including several key regions of E2 neutralizing antibody recognition. The antibody concentration used in epitope mapping was optimized by a dose-dependent study employing 0.005–2 μg/ml against wt 1a H77C E1E2 and a dose was chosen at 50% of maximum binding and in the linear portion of the binding curve, 212.1 at 2 μg/ml, 212.10 at 1 μg/ml, 212.15 at 0.5 μg/ml and 212.25 at 2 μg/ml (S9 Fig). Control antibodies, HC-1, HC-11, CBH-5, HC84.26 and CBH-7 were at 1 μg/ml. 212.1.1 and 212.10 showed binding reduction patterns similar to HC-1 and HC-11, including two key domain B contact residues at G530A and D535A [13]. The involvement of binding to residues within aa 529–540 is central to domain B, and these residues are also key for antibodies targeting E2 antigenic region 3 (AR3; HMAbs AR3A, AR3B, AR3C, AR3D), as shown by recent global epitope mapping study [30]. The involvement of binding to 440–445 and without binding to aa 529–540 is central to domain D [11]. Thus, 212.1.1 and 212.10 HMAbs are within the domain B cluster, though their mapping shows differential binding determinants, including residue F442 where alanine substitution does not disrupt 212.1.1 binding but results in over 80% loss of 212.10 binding. Notably, AR3 antibodies also lose binding when F442 is mutated to alanine [30, 31], suggesting greater similarity to HC-11 and 212.10 than to HC-1 and 212.1.1. As expected, these patient 212 antibodies blocked E2 interaction with CD81 (S10 Fig). Pre-incubation of 1a H77C E2 glycoproteins with either 212.1.1 or 212.10 reduced E2 binding to CD81, as observed with a control domain B antibody, HC-11. However, 212.9 is likely a domain B antibody as evidenced by its ability to block HC-11 and 212.10 binding to autologous 1b E2 by > 60% (S11 Fig).

HMAbs 212.15 and 212.25 blocked mainly CBH-7, an antigenic domain C antibody (S3 Table). Epitope mapping confirmed that their epitopes have contact residues within aa 544–549, as shown by greater than 60% reduction in binding to P544A, P547A and W549A (Fig 6). These patterns are similar to CBH-7. In addition, both antibodies blocked E2 binding to CD81 (S10 Fig). However, 212.15 and 212.25 HMAbs have a critical binding determinant at residue R639, which is not shared with CBH-7, but is shared with previously described antibody AR5A that targets the E1E2 heterodimer [32]. Taken together, the neutralizing antibody response in the individual 300212 during the acute phase of spontaneous clearance of a second virus is directed mainly at two antigenic clusters, B and C.

### Broadly neutralizing antibodies to epitopes encompassing aa 523–527 do not impair HCV cell-to-cell transmission

Using an established experimental assay [33], we assessed whether HMAbs 212 impaired viral cell-to-cell transmission in HCV Jc1-infected Huh7.5.1 cells. As shown in S12 Fig, the HMAbs did not have a major inhibitory effect on cell-to-cell transmission of HCV Jc1 strain. It should be noted that HMAb 212.10 neutralized the cell-free J6 HCVcc that is derived from the same isolate as Jc1, while 212.1.1 could not neutralize cell-free J6 HCVcc (Table 3) [15, 34].

### HMAb germline and CDR sequences

To assess sequence features underlying the HMAbs 212 and antibodies targeting domains B and C, germline genes, CDR3 amino acid sequence, and levels of somatic hypermutations (SHMs) were compared with previously described domain B and C HMAbs (Table 4) [13, 35–38]. HMAb 212 germline percent identities are at or above 89% for heavy chains variable regions (Vh), and over 95% for light chain variable regions (Vl), in concordance with a previous study where broadly neutralizing antibodies from donors who spontaneously cleared HCV were observed to have few SHMs [38] (one of those HMAbs, HEPC3, is included in Table 2). One feature of the 212 and other domain B and C HMAbs in Table 4 is that all of their heavy chains share the same germline gene, IGHV1-69, despite variability in CDR H3 sequence and length, and light chain germline genes. This preferential usage of IGHV1-69 gene in E2 HMAbs was noted previously [38–40] and may suggest a shared set of solutions for targeting these conformational epitopes on E2, possibly facilitated by hydrophobic germline residues in the CDR H2 loop which underlie IGHV1-69 gene usage in influenza hemagglutinin stem antibodies [41].

**Table 4:** 

		Heavy			Light			
HMAb	Domain^1^	Vh	% ID^2^	CDR3	Vl	% ID^2^	CDR3	Reference^3^
HC-1	B	1–69	90	AKVLQVGGNLVVRPL	κ3–20	93	HQYGNSPQT	
HC-11	B	1–69	92	AMEVPGFCRGGSCSGYMDV	κ3–20	96	QQYGSSPIT	
AR3C	B	1–69	86	VRSVTPRYCGGGFCYGEFDY	κ3–15	95	QQYYRSPLT	[36, 38]
AT12-009	B	1–69	87	VTSLSEPIPRSCRGGRCYSGPFDAFGV	κ3–15	94	QQYNNWPLT	[37, 67]
AT12-010	B	1–69	92	GRDRAPRLCSGGRCHSPPDH	κ1–12	93	LQTNTFPYT	[37, 67]
AT13-021	B	1–69	93	ARELIGYCTGGNCYSFGDF	κ3–15	97	HQYNTWPRG	[37, 67]
CBH-5	B	1–69	89	ARPHGDSSGIYRRPLDY	κ1–5	93	QYYNNFSGT	
212.1.1	B	1–69	93	AGVREGMAAISGKNAFDI	κ3–20	97	QQYNNWPPS	
212.10	B	1–69	89	ATDRMRDDSTLFRDSHFDN	κ1–39	98	QQSYSTPYT	
HEPC3	B	1–69	95	ARDGVRYCGGGRCYNWFDF	κ1–39	96	QQSHSTVRT	[38, 57]
HEPC74	B	1–69	92	ARDLLKYCGGGNCHSLLVDP	κ1–5	96	QHYNTYLFT	[38, 57]
AT12-011	C	1–69	88	ARHDYFWGTPLDI	κ6–21	97	HQSYNLPRT	[37, 67]
CBH-7	C	1–69	92	ARRGYIYGSPFDY	κ1–39	96	QQSYSPLLT	
212.15	C	1–69	92	TRRSHYYGSGLDS	κ1–12	96	QQSYSTPYT	
212.25	C	1–69	93	ARGGDLYGSGPLYYYYGMDV	κ3–20	96	QQYDNLPPL	
HC84.26	D	1–69	91	ARGPLSRGYYDY	λ3–21	99	QVWDSSSVV	

Heavy chain CDR3 cysteine residue pairs are highlighted in red.

^1^E2 antigenic domain targeted by the antibody, based on binding competition and epitope mapping.

^2^Percent nucleotide sequence identity to germline gene.

^3^Reference for antibody and sequence information, for HMAbs reported in other studies.

Several of the domain B HMAbs also share a distinctive double-cysteine and double-glycine motif (CxGGxC) in the context of CDR H3 loops ranging from 19 and 27 residues long. Additionally, in four out of five cases, this motif is preceded by a proline residue (i.e., PxxCxGGxC). The precise cysteine-glycine organization of these HMAbs may be critical for antibody affinity and neutralization; for the AR3C HMAb, which has been structurally characterized in complex with E2, the cysteine residues form a disulfide bond, likely stabilizing the CDR H3 loop in a β-hairpin conformation which makes numerous key hydrophobic contacts and hydrogen bonds with E2 [42]. A search of the set of experimentally determined antibody structures in the PyIgClassify database [43] (June 2018 release) for other CDR H3 loops containing the CxGGxC motif identified two motif-containing CDRs out of 2172 unique CDR H3 sequences. These correspond to the broadly neutralizing antibody F10 which targets the influenza hemagglutinin stem and also includes the IGHV1-69 germline gene (PDB code 3FKU) [44], and the 8f9 antibody which binds a peptide antigen from human cytomegalovirus (PDB codes 3EYF, 3EYO) [45]. In both cases, intra-loop disulfide bonds are present, as with AR3C, suggesting that this is a shared mechanism to stabilize CDR H3 loops, and that other domain B HMAbs with this motif are likewise disulfide-stabilized, possibly in an AR3C-like β-hairpin conformation.

### Neutralizing antibody response to less immunogenic regions on HCV E2

A conserved region on E2, encompassing aa 412–423 (designated as antigenic domain E), mediates broadly neutralizing antibodies to linear epitopes, but is known to be of lower immunogenicity than other regions [12, 46]. An adjacent region, encompassing aa 434–446, is also likely to be less immunogenic, participates in forming conformational epitopes (designated as antigenic domain D) and mediates broad virus neutralization [11]. Although domain D antibodies are directed against conformational epitopes, a significant number of these antibodies also bind to a linear peptide encompassing aa 434–446 on E2. Thus, to assess whether the antibody response in individual 300212 included antibodies to domains D and E, sequential serum samples from weeks 21 to 182 were tested in serial dilutions for binding to synthetic peptides encompassing aa 410–425 and aa 434–446. Minimal reactivity was observed against aa 410–425 with a significant dropoff in binding (>0.4 O.D.) after 1:1000 serum dilutions for the tested timepoints (Table 5). Significantly stronger reactivities were observed against aa 434–446 at multiple timepoints, weeks 76, 122 and 123, where >0.4 O.D. at 1:5000 or greater in dilution (Table 6). Interestingly, binding by serum antibodies at week 21 to aa 434–446 was observed. This finding was surprising in that antigenic domain B and C antibodies (e.g. 212.1.1 and 212.15, Fig 4A and 4B) were not present at this timepoint. To characterize more definitively the antibody response to antigenic domain D and because these antibodies are to conformational epitopes, the ability of sequential serum samples to block HMAbs to D and E epitopes was tested (Fig 4C and 4D). As expected, no inhibition was observed against HC33.1 (a domain E HMAb [12]) but dose-dependent inhibition was observed against HC84.27 (a domain D HMAb [11]). Maximum inhibition at 1:100 serum dilution against HC84.27 was highest at week 76, which then persisted throughout the remaining of the course of clinical observation (Fig 4C). In contrast to serum binding at 1:100 dilution to peptide aa 434–446 at week 21 (Table 6), the serum at this timepoint did not inhibit HC84.27 (Fig 4C). It is possible that this discrepancy is due to different assay formats having differential sensitivity to detect the presence of domain D antibodies, with a direct binding assay being more sensitive, as shown in Table 6. It is also possible that peptide aa 434–446 is recognized by other antibodies to mainly linear epitopes [47].

**Table 5:** Serum antibody binding to synthetic peptide aa410-425.

	Serum Dilution						O.D.
Timepoint	100	500	1000	5000	10000		>2
21	**0.03**	**0.05**	**0.08**	**0.09**	**0.09**		**1–2**
76	**2.19**	**2.09**	**1.44**	**0.19**	**0.03**		**0.5–1**
122	**1.82**	**0.78**	**0.49**	**0.00**	**0.00**		**0.2–0.5**
123	**1.78**	**0.79**	**0.48**	**0.04**	**0.00**		
135	**1.81**	**0.82**	**0.48**	**0.13**	**0.12**		
150	**2.07**	**0.67**	**0.39**	**0.03**	**0.00**		
182	**1.83**	**0.56**	**0.46**	**0.08**	**0.04**		

**Table 6:** Serum antibody binding to synthetic peptide aa434-446.

	Serum Dilution						
Timepoint	100	500	1000	5000	10000		O.D.
21	**1.00**	**0.37**	**0.23**	**0.06**	**0.03**		**> 2**
76	**2.52**	**2.46**	**2.02**	**0.78**	**0.46**		**1–2**
122	**2.49**	**1.87**	**1.21**	**0.42**	**0.22**		**0.5–1**
123	**2.57**	**1.87**	**1.29**	**0.44**	**0.23**		**0.2–0.5**
135	**2.44**	**1.62**	**1.20**	**0.38**	**0.20**		
150	**2.21**	**1.50**	**1.08**	**0.36**	**0.20**		
182	**2.28**	**1.46**	**1.17**	**0.31**	**0.17**		

### Viral isolate sequences

**Fig 7:**
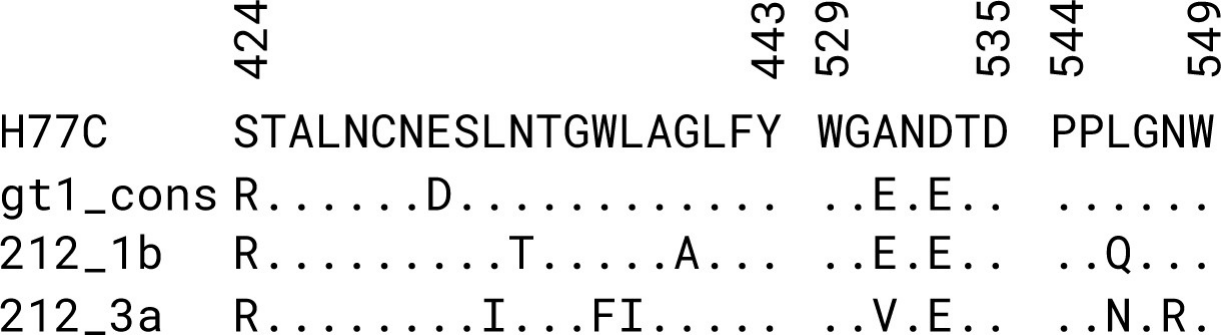
Sequences of viral isolates from individual 212. A multiple sequence alignment of E1E2 amino acid sequences from single 212 1b and 3a clones was produced, with H77C and genotype 1 consensus for reference, using MAFFT software [60]. The genotype 1 consensus protein sequence was downloaded as part of the 2014 reference sequence set from the LANL HCV database [60]. Three regions including common determinants of domain B, D and C HMAbs, corresponding to Regions 1–3 in Fig 6, are shown. "." denotes identical amino acid residue at that position compared with the top sequence (H77C).

To determine the presence of any particular sequence features underlying elicitation of broadly neutralizing antibodies and viral clearance, 212 1b and 212 3a E1E2 sequences were aligned along with H77C and genotype 1a consensus reference sequences, with the latter sequence obtained from the Los Alamos National Laboratory HCV database [48] (Fig 7). Inspection of three regions that contain binding determinants of HMAbs 212 and domain B, C and D antibodies did not show major sequence changes for the 212 sequences, in particular for the 212 1b sequence, which was present at a timepoint that would potentially influence selection and maturation of 212 HMAbs. It is unclear whether specific variations (e.g. G440A) or combinations thereof in the 212 1b sequence possess any features leading to antibody elicitation, though further investigation of these and other viral sequences may provide avenues to optimize E2 and E1E2-based vaccine immunogens.

## Discussion

The majority of neutralizing antibodies against HCV are directed against the E2 envelope glycoprotein, because E2 directly interacts with the HCV co-receptors, scavenger receptor class B type 1 (SR-B1) [49] and the tetraspanin CD81 [50] during virus entry. There is recent evidence that E1E2 heterodimers, and not E2 alone, interacts with a third co-receptor, the tight junction protein Claudin-1 [51]. While effective neutralizing antibodies are directed at the HVR1 on E2, this region is associated with mutations leading to rapid viral escape without compromising viral fitness [24, 28]. Based on the isolation and characterization of HCV HMAbs from B cells of patients with chronic HCV infections, the majority of broadly neutralizing antibodies recognize conformational epitopes on E2 and inhibit E2 binding to CD81 [26]. Cross-competition analyses delineate at least four immunogenic clusters of overlapping conformational epitopes with distinct properties [11, 27]. Non-neutralizing HMAbs fall within one cluster, which is designated as antigenic domain A. It is probable that this cluster of conformational epitopes and other non-neutralizing determinants are highly immunogenic and account for a substantial portion of the antibody response to E2 [52]. Neutralizing HMAbs segregate into three clusters of conformational epitopes, antigenic domain B, C and D. A fifth cluster of linear epitopes mediating neutralization, domain E, located at aa 412–423 on E2, has been identified by both murine and human monoclonal antibodies (reviewed in [26]). As the human antibodies isolated from chronically infected subjects co-existed with viremia, a minimal role for neutralizing antibodies in viral clearance has been the conventional view. Nonetheless, accumulated findings have now provided strong support for neutralizing antibodies facilitating spontaneous viral clearance during acute infection [7, 8]. The question that has been addressed in the current detailed study of the antibody response in an individual who repeatedly cleared HCV infections of different genotypes is whether the specificity of the response is similar or dissimilar to the response observed in individuals that developed chronic infections. Our findings are in agreement with the recent report of other antibodies isolated from individuals during acute HCV infections that spontaneously resolved infections. Their isolated HMAbs were similar to those isolated during chronic infection [38].

Overall, the studied individual showed a pattern of cross-genotype reactive neutralizing antibodies increasing in titers from the viremic phase to spontaneous clearance of the two first HCV infections. This pattern is consistent with earlier studies on the timing of appearance of broadly neutralizing antibodies response correlating with spontaneous viral clearance [7, 8]. However, the individual did get re-infected in spite of having a robust antibody response at week 76 after the first documented infection (Table 1). Contributing to viral clearance are most likely robust CD4+ and CD8+ T cell responses that were temporally observed in the studied individual during the primary and repeated infections (*personal communication and manuscript in preparation*, *A*. *Lloyd*). We believe that both cellular and humoral immunity contributed to the repeated episodes of spontaneous clearance. As for the specificities of the antibody responses, the first indication that these antibodies are directed to conformational epitopes is based on the decrease in serum antibody binding against denatured autologous and heterologous E1E2 recombinant proteins. Autologous and heterologous E2 proteins were employed to isolate a panel of HMAbs. Of 14 antibodies that neutralized at least one of the tested isolates, eight neutralized autologous 212 1b HCVpp and these eight also neutralized at least one of the heterologous isolates. No neutralizing antibodies directed at HVR1 were isolated. This is somewhat surprising since HVR1 is immunodominant. A likely contributing factor is that these antibodies were isolated from a B cell response associated with the second infection with a different isolate, and we screened with autologous E2 from the first infection. A second possible factor is that the subject reported long periods of at least daily injection drug use and sharing of these injections with other users. It is possible that during the 24 months from the initial infection (week 21) to the second infection (week 122), the subject had other undocumented episodes of HCV infection. Thus, the profile of the antibody response at week 123 may reflect multiple exposures to HCV that are associated with repeated viral clearance. This may also pertain to the interval from weeks 123 to 182, underpinning the high serum antibody binding and neutralizing titers maintained during this period.

In the panel of HMAbs that were isolated, two antibodies, 212.1.1 and 212.9, accounted for more than 50% of the total clones analyzed. These two antibodies and a third, 212.10, are within a highly immunogenic cluster, designated as domain B. Antibodies to overlapping epitopes within this antigenic domain account for the majority of described broadly neutralizing antibodies [26], and here we found that 212.10 indeed neutralized strains representing 8 subtypes of genotypes 1–6. While many of these antibodies exhibit broad neutralization, they can be associated with viral escape, with and without compromised viral fitness [53]. Both 212.1.1 and 212.10 appear to bind to overlapping epitopes involving aa 523–527, a region on E2 with a possible role in cell-to-cell transmission [54]. However, no inhibition was observed with either antibody. HMAb 212.15 and 212.25 represent antibodies to another cluster of overlapping epitopes, antigenic domain C [27]. Epitope mapping of these antibodies was similar to CBH-7 (a domain C antibody) that was previously isolated from a chronically infected patient. Antibodies to both of B and C domains remained at relatively stable titers from weeks 76–182, although somewhat higher at week 76 (Fig 4A and 4B). The implication being that by week 76, optimal *in vivo* affinity maturation of these antibodies had occurred, and there is no significant bias in the induction of antibodies to these domains. Taken together, these results suggest that antigenic domain B and C antibodies contributed to the protective immunity in this individual. While this immunity did not prevent reinfection, it did potentially prevent progression to chronic infection. Additional studies are required to determine whether these antibodies elicited during acute infection are associated with viral escape, as demonstrated for other HMAbs [11, 25, 53, 55].

The studied individual with acute HCV infections also developed antibodies against antigenic domain D. Although domain D antibodies are directed to conformational epitopes, some of these antibodies also bind to synthetic peptides encompassing aa 434–446 [11]. Sequential serum reactivity to this peptide and inhibition of the binding of a labelled domain D HMAb to E2 indicate the presence of domain D-specific antibodies in these sera. Finding antigenic domain D antibodies is unusual in that this region is of lower immunogenicity, as indicated by the identification of HMAbs to this domain only after eliminating the detection of antigenic domains A and B antibodies [11]. This domain is known to have highly conserved overlapping epitopes and the associated antibodies have the broadest reactivity among diverse HCV genotypes and subtypes, as compared to domains B and C antibodies. Not only are these part of the antibody response in the individual reported here, but the antibodies may appear at the earliest time point at week 21, as detected by direct serum antibody binding to aa 434–446, when antibodies to antigenic domain B and C were not present. Although serum neutralizing activity titers were less than 100 at this timepoint, the binding studies clearly indicated the presence of domain D antibodies, albeit at low levels. One possible interpretation of these findings is that protective B cell immunity is associated with the early induction of neutralizing antibodies to antigenic domain D. To support this observation, other studies will be needed to remove the possibility that aa 434–446 is recognized by non-domain D antibodies in this individual. Additional studies will be required in other individuals that naturally cleared HCV infections to confirm this observation on early induction of these protective antibodies. Interestingly, the study subject carries the *IL28B* rs12979860 polymorphism that has been associated with spontaneous viral clearance [10, 56] raising the suggestion that this innate immune response genotype may be linked to development of effective neutralizing antibody responses.

The monoclonal antibodies described in this study underscore the importance of features of the human antibody repertoire, including the IGHV1-69 germline gene, in targeting HCV E2. Others recently isolated three IGHV1-69 HMAbs that target antigenic domain B (two of these, HEPC3 and HEPC74, are noted in Table 4) from two individuals that spontaneously cleared HCV [38], while intriguingly, a fourth HMAb described in the same study with the IGHV1-69 germline gene had its binding mapped to residues in HVR1 [38]. Likewise, we found that antigenic domain C, which is distinct from antigenic domain B [27], is targeted by antibodies with the IGHV1-69 germline gene, including two HMAbs described here. Future studies can address the mechanistic and structural basis underlying the usage of this gene by antibodies targeting these distinct sites. Interestingly, HMAbs HEPC3 and HEPC74 had their structures determined in complex with E2, and the HMAb AR3A-E2 complex structure was described in a separate recent study [40]; all feature disulfide-stabilized HCDR3 loops forming critical interactions with antigenic domain B [57], in a similar manner as AR3C [36]. This supports the HCDR3 putative disulfide bond and common mode of E2 targeting for HC-11 and other antigenic domain B HMAbs with the cysteine pair motif noted in Table 4.

Collectively, our results indicate that the development of a successful B cell vaccine will require an understanding of the timing and affinity maturation of the humoral immune response. In addition, there appears to be no advantages to study individuals with spontaneous clearance as sources for B cells for the isolation of broadly neutralizing antibodies compared to patients with chronic infection.

## Materials and methods

### Ethics statement

Human research ethics approvals were obtained from Human Research Ethics Committees of Justice Health (reference number GEN 31/05), New South Wales Department of Corrective Services (05/0884), and the University of New South Wales (05094, 08081), all located in Sydney, Australia. Written informed consent was obtained from the adult participants.

#### Cell culture, antibodies, virus and reagents

HEK-293T cells were obtained from the ATCC. The Huh7.5 cells, the 9E10 antibody and J6/JFH1 recombinant virus (HCVcc) were generously provided by Dr. C. Rice (Rockefeller University). Dr. T. Wakita (National Institute of Infectious Diseases, Japan) and Dr. J. K. Ball (University of Nottingham) generously provided respectively the 2a JFH1 HCVcc and a panel of genotype 1–6 E1E2 constructs. The CD81 large extracellular loop fused to glutathione *S*-transferase was provided by Dr. S. Levy (Stanford University). Anti-CD81 (JS-81) was from BD Biosciences (Heidelberg, Germany) and anti-core C7-50 was from Abcam (Cambridge, UK).

#### Viral analysis

Qualitative HCV RNA detection was performed as described [58]. Viral genotyping and testing for mixed infection was conducted as described [59]. The HCV genotype 1 E1 and E2 consensus protein sequences were downloaded from the LANL HCV database (2014 genotype reference aligned sequences) [48], and E1E2 amino acid sequences were aligned using MAFFT software [60].

#### Generation and selection of yeast display HCV-E2-scFv

The methods for generating the yeast scFv displayed library, selection of HCV E2-specific scFv and production of scFvs and IgG_1_ HMAbs have been described previously in details [11]. Selection was by a combination of employing 212 1b and H77C recombinant E2 proteins. Individual scFv clones reactive to either E2 by flow analysis were sequenced; soluble scFv and full-length IgG_1_ were produced and purified as described [11].

#### Neutralization assays (HCVcc and HCVpp)

HCVcc neutralization was measured with a modified version of an FFU assay as described previously [11]. Serial dilutions of HMAbs and HCVcc (containing a volume of viral stock with a readout of approximately 3000 FFU) were pre-incubated for 1h. The virus-HMAb mixture was added to Huh7.5 cell monolayers and incubated for 3h. Fresh medium were added to the infected cells after washing and incubated for 72h. Cells were then washed and lysed with lysis buffer in the presence of protease inhibitors. Lysate was transferred to GNA-coated plate and incubated for 1h. Bound E2 was detected by a HMAb to E2, CBH-5 [27], and detected by HRP conjugated anti-human IgG by ELISA, as described below. The infectivity reduction was determined by the relative O.D. (450/570 nm) in the presence and absence of antibody and was expressed as a percentage of neutralization. Production and neutralization of HCV retroviral pseudotype particles expressing E1E2 (HCVpp) were as described [61].

#### Testing of 212 HMAbs neutralization against Core-NS2 HCVcc virus panel

The panel represented HCV strains of genotypes 1–6 [62]. We performed HCVcc neutralization as outlined in Springer Protocols [14]. We plated 7x10^3^ Huh7.5 cell per well in poly-D-lysine 96-well plates and incubated for 24 hours. Next day, a volume of virus stock corresponding to a read-out of 20–200 FFU per well were incubated in quadruplicates with a dilution series of 212 monoclonal antibodies or relevant positive and negative control antibodies. Virus-antibody mixes along with eight replicates of virus only were incubated for 1 hour at 37°C, then added to Huh7.5 cells and incubated for 4 hours at 37°C and 5% CO_2_. Subsequently, the cells were washed, and fresh medium was added prior to incubation for a total infection time of 48 hours. Cells were fixed and stained using 9E10 antibody as described previously [25], and the number of FFUs were counted using an ImmunoSpot Series 5 UV Analyzer as described [63]. The data were normalized to 8 replicates of virus only and analyzed using four-parameter curve fitting in GraphPad Prism 7.02. Virus stocks of all tested HCV recombinants had confirmed sequences of the encoded E1E2 envelope proteins.

### Enzyme-linked immunoassays

#### a. Clinical HCV assays

HCV-specific antibody detection during the prospective study on subject’s serum samples was conducted as described below for binding to native and denatured E1E2 glycoprotein.

#### b. Serum competition assay

Serum samples at specified dilutions were tested for their ability to block the binding of selected HCV HMAbs-conjugated with biotin in a GNA-captured E1E2 glycoproteins ELISA, as described [11].

#### c. Binding to native and denatured E1E2 glycoprotein

A standard ELISA was employed to compare serum antibody or HMAb binding to native and denatured HCV E1E2 glycoproteins as described [11]. Briefly, recombinant autologous 212 1b and H77C E1E2 lysates were either left untreated or denatured by incubation with 0.5% sodium dodecyl sulfate and 5 mM dithiothreitol for 15 min at 56°C. After treatment, the proteins were diluted 1:5 in BLOTTO and captured by pre-coated GNA wells. After washing and blocking, bound proteins were incubated with diluted serum diluted or HMAb as indicated. Bound antibodies were detected as described.

#### d. Binding to a panel of genotype 1–6 E1E2 glycoproteins

A standard ELISA was employed as outlined above for (**c**) binding to native E1E2 glycoprotein.

#### e. Blocking of E2 binding to CD81

Selected HMAbs at specified concentrations were incubated for 20 min at 4°C with recombinant E2. The HMAbs-E2 mixture was then added to ELISA plates pre-coated with soluble CD81-LEL that were blocked with 2.5% BSA and 2.5% normal goat serum in 0.1% Tween-PBS. Bound E2 was detected with biotinylated-CBH-4D, a non-neutralizing HCV HMAb as described [27].

#### Epitope mapping

Epitope mapping was performed using alanine substitution mutants by ELISA. Alanine substitution mutants were constructed as described [53] in plasmids carrying the H77C E1E2 coding sequence (GenBank accession no. AF011751.1). The mutated constructs were designated X#Y, where # is the residue location in H77C, X denotes the single-letter code for the H77C amino acid, and Y denotes the altered amino acid.

#### HCV cell-cell transmission assay

The effect of antibodies on HCV cell-to-cell transmission was assessed as described [33].

#### Clustering

Hierarchical clustering of alanine scanning data was performed as previously described [29] using R (http://www.R-project.org/), after transforming binding percentage data to log ratios, with binding values of 0% set to 0.5% to permit log calculation. E2 positions and antibodies were compared using Euclidean and correlation-based distances, respectively, and clustered using Ward’s minimum variance method. Positions that lacked binding data for one or more HMAbs were removed prior to clustering. Cluster p-values were computed using the approximate unbiased method in the pvclust R package [64], with 10,000 bootstrap replicates. The unrooted tree was generated using the Analyses of Phylogenetics and Evolution (APE) package [65] in R.

#### Antibody sequence analysis

Antibody nucleotide sequences were analyzed using IMGT/V-QUEST web server [66] to determine percent identity to Vh and Vl germline genes.

#### Study approval

All protocols were approved by the Institutional Review Board of Stanford University. Ethical approvals were obtained from Human Research Ethics Committees of Justice Health (reference number GEN 31/05), the New South Wales Department of Corrective Services (reference number 05/0884), and the University of New South Wales (reference numbers 05094, 08081, 13237, 09075, 14170), and written informed consent was obtained from the study adult participant.

## Supporting information

S1 Fig Individual 212 serum neutralization titers against heterologous genotype 1 HCVpp. The % neutralization (*y-axis*) was plotted against the log^10^ serum dilution (*x-axis*) for all defined time points listed in the figure legends. A non-linear regression analysis (log inhibitor vs normalized response) was performed in GraphPad PRISM v7.02 to calculate the estimated inhibitory concentration required to neutralize 50% of the virus (IC_50_). The % neutralization was calculated by comparing the RLU of healthy serum to the RLU of the tested serum (1-[RLU test serum]/[RLU healthy serum]).
(PDF)

S2 Fig Individual 212 serum neutralization titers against heterologous genotype 2 HCVpp. The % neutralization (*y-axis*) was plotted against the log^10^ serum dilution (*x-axis*) for all defined time points listed in the figure legends. A non-linear regression analysis (log inhibitor vs normalized response) was performed in GraphPad PRISM v7.02 to calculate the estimated inhibitory concentration required to neutralize 50% of the virus (IC_50_). The % neutralization was calculated by comparing the RLU of healthy serum to the RLU of the tested serum (1-[RLU test serum]/[RLU healthy serum]).
(PDF)

S3 Fig Individual 212 serum neutralization titers against heterologous genotype 3 HCVpp. The % neutralization (*y-axis*) was plotted against the log^10^ serum dilution (*x-axis*) for all defined time points listed in the figure legends. A non-linear regression analysis (log inhibitor vs normalized response) was performed in GraphPad PRISM v7.02 to calculate the estimated inhibitory concentration required to neutralize 50% of the virus (IC_50_). The % neutralization was calculated by comparing the RLU of healthy serum to the RLU of the tested serum (1-[RLU test serum]/[RLU healthy serum]).
(PDF)

S4 Fig Individual 212 serum neutralization titers against heterologous genotypes 4–6 HCVpp. The % neutralization (*y-axis*) was plotted against the log^10^ serum dilution (*x-axis*) for all defined time points listed in the figure legends. A non-linear regression analysis (log inhibitor vs normalized response) was performed in GraphPad PRISM v7.02 to calculate the estimated inhibitory concentration required to neutralize 50% of the virus (IC_50_). The % neutralization was calculated by comparing the RLU of healthy serum to the RLU of the tested serum (1-[RLU test serum]/[RLU healthy serum]).
(PDF)

S5 Fig Neutralization of HCVcc recombinants with Core-NS2 from genotype 1 HCV isolates. Virus stocks of the indicated genotypes 1a and 1b HCV Core-NS2 recombinants were subjected to dose-response FFU reduction neutralization assays using dilution series of the antibodies (A) 212.1.1, (B) 212.10, (C) 212.25, (D) R04, or (E) HC84.27 in quadruplicates with 8 wells of virus only. Following a total of 48 hours infection the cells were immuno-stained and the number of FFUs per well were counted as described in Materials and Methods. Error bars represent standard error of the mean of four replicates normalized to 8 replicates of virus only. The data was analyzed using four-parameter curve-fitting to obtain a sigmoidal dose-response curve, permitting the interpolation of an IC_50_ value (Graphpad PRISM 7.02). J4 and H77_ΔHVR1_ gave fewer than 20 FFUs/well in virus only wells against all antibodies.
(PDF)

S6 Fig Neutralization of HCVcc recombinants with Core-NS2 from genotype 2 HCV isolates. Virus stocks of the indicated genotypes 2a and 2b HCV Core-NS2 recombinants were subjected to dose-response FFU reduction neutralization assays using dilution series of the antibodies (A) 212.1.1, (B) 212.10, (C) 212.25, (D) R04, or (E) HC84.27 in quadruplicates with 8 wells of virus only. Following a total of 48 hours infection the cells were immuno-stained and the number of FFUs per well were counted as described in Materials and Methods. Error bars represent standard error of the mean of four replicates normalized to 8 replicates of virus only. The data was analyzed using four-parameter curve-fitting to obtain a sigmoidal dose-response curve, permitting the interpolation of an IC_50_ value (Graphpad PRISM 7.02).
(PDF)

S7 Fig Neutralization of HCVcc recombinants with Core-NS2 from genotype 3 HCV isolates. Virus stocks of the indicated genotype 3a HCV Core-NS2 recombinants were subjected to dose-response FFU reduction neutralization assays using dilution series of the antibodies (A) 212.1.1, (B) 212.10, (C) 212.25, (D) R04, or (E) HC84.27 in quadruplicates with 8 wells of virus only. Following a total of 48 hours infection the cells were immuno-stained and the number of FFUs per well were counted as described in Materials and Methods. Error bars represent standard error of the mean of four replicates normalized to 8 replicates of virus only. The data was analyzed using four-parameter curve-fitting to obtain a sigmoidal dose-response curve, permitting the interpolation of an IC_50_ value (Graphpad PRISM 7.02).
(PDF)

S8 Fig Neutralization of HCVcc recombinants with Core-NS2 from HCV isolates of genotypes 4–6. Virus stocks of the indicated genotypes 4a, 5a, or 6a HCV Core-NS2 recombinants were subjected to dose-response FFU reduction neutralization assays using dilution series of the antibodies (A) 212.1.1, (B) 212.10, (C) 212.25, (D) R04, or (E) HC84.27 in quadruplicates with 8 wells of virus only. Following a total of 48 hours infection the cells were immuno-stained and the number of FFUs per well were counted as described in Materials and Methods. Error bars represent standard error of the mean of four replicates normalized to 8 replicates of virus only. The data was analyzed using four-parameter curve-fitting to obtain a sigmoidal dose-response curve, permitting the interpolation of an IC_50_ value (Graphpad PRISM 7.02). ED43 gave fewer than 20 FFUs/well in virus only wells against 212.10 and 212.25 and SA13 gave more than 200 (but no more than 210) FFUs/well in virus only wells against 212.1.1, R04, and HC84.27.
(PDF)

S9 Fig Dose-dependent binding of HMAbs 212 to H77C E1E2. Recombinant H77C 1a E1E2 lysates were captured by pre-coated GNA wells. After washing and blocking, bound proteins were incubated with each indicated 212 HMAb at 0.005–2 μg/ml (*x*-axis) for 30 minutes. After washing, bound antibodies were detected as described in Materials and Methods. The *y*-axis shows the mean optical density values for triplicate wells, the mean of two experiments ± SD.
(PDF)

S10 Fig Inhibition of E2 binding to CD81-LEL by HMAbs 212. H77C 1a recombinant E1E2 lysate containing 1 μg/ml E2 was incubated with each test HMAb at 10 μg/ml. The antibody-antigen complex was then added onto CD81-LEL-precoated wells. Detection of E2 bound to CD81-LEL was measured with biotinylated CBH-4D [13, 35–38]. HC-11 was used as a positive control and R04 as a negative control (a HMAb to HCMV). Inhibition of binding is expressed as percent inhibition (*y-axis*). Experiments were performed twice in triplicate. Error bars indicate one standard deviation from the mean.
(PDF)

S11 Fig HMAb 212.9 is against an epitope within antigenic domain B. Recombinant autologous 1b E1E2 lysates were captured by pre-coated GNA wells. After washing and blocking, bound proteins were incubated with each indicated 212 blocking antibody at 20 μg/ml (*x*-axis), a control antigenic domain B HMAb, HC-11, and a no antibody control for 30 minutes. After washing, labeled 212.9 HMAb at 2 μg/ml was added. Bound 212. 9 HMAb was detected as described in Materials and Methods. The *y*-axis shows the percent competition by each blocking antibody, the mean of two experiments ±SD that were performed in triplicates.
(PDF)

S12 Fig HMAbs 212 do not inhibit cell-cell transmission. (A) Approach of HCV cell-cell transmission experiments. HCV Huh7.5.1 producer cells cultured with naïve Huh7.5-GFP target cells were incubated with control or anti-HCV HMAbs (100 μg/ml) in the presence of anti-HCV IgG (50 μg/ml) to block cell-free transmission similar as described [33]. Cell-to-cell transmission was determined by quantification of HCV+ GFP+ target cells using immunostaining and flow cytometry. (B) HCV cell-cell transmission indicated as percentage of HCV-infected Huh7.5-GFP target cells is shown as histogram. Means +/- SD from two independent experiments performed in duplicate are shown. Incubation of cells with anti-CD81 MAb served as positive control.
(PDF)

S1 Table Chronology of multiple HCV infections. HCV RNA was detected as described in Materials and Methods.
(PDF)

S2 Table Antibody binding to different HCV genotypes and autologous isolates. A standard ELISA against of native E1E2 glycoproteins was performed as described in Materials and Methods.
(PDF)

S3 Table Competition of 212 antibody binding to E2. This study was performed as described in Materials and Methods.
(PDF)

## References

[1] Mohd Hanafiah K, Groeger J, Flaxman AD, Wiersma ST. Global epidemiology of hepatitis C virus infection: new estimates of age-specific antibody to HCV seroprevalence. Hepatology. 2013;57(4):1333–42. 10.1002/hep.26141 . 23172780

[2] WHO. Global hepatitis report, 2017. 2017:83. Epub April 2017.

[3] Liang TJ, Ghany MG. Current and future therapies for hepatitis C virus infection. N Engl J Med. 2013;368(20):1907–17. Epub 2013/05/17. 10.1056/NEJMra1213651 . 23675659 PMC3893124

[4] Bukh J. The history of hepatitis C virus (HCV): Basic research reveals unique features in phylogeny, evolution and the viral life cycle with new perspectives for epidemic control. J Hepatol. 2016;65(1 Suppl):S2–S21. 10.1016/j.jhep.2016.07.035 . 27641985

[5] Edlin BR. Access to treatment for hepatitis C virus infection: time to put patients first. Lancet Infect Dis. 2016;16(9):e196–e201. 10.1016/S1473-3099(16)30005-6 . 27421993

[6] Grebely J, Page K, Sacks-Davis R, van der Loeff MS, Rice TM, Bruneau J, et al The effects of female sex, viral genotype, and IL28B genotype on spontaneous clearance of acute hepatitis C virus infection. Hepatology. 2014;59(1):109–20. 10.1002/hep.26639 23908124 PMC3972017

[7] Pestka JM, Zeisel MB, Blaser E, Schurmann P, Bartosch B, Cosset FL, et al Rapid induction of virus-neutralizing antibodies and viral clearance in a single-source outbreak of hepatitis C. Proc Natl Acad Sci U S A. 2007;104(14):6025–30. Epub 2007/03/30. 0607026104 [pii] 10.1073/pnas.0607026104 17392433 PMC1851610

[8] Osburn WO, Snider AE, Wells BL, Latanich R, Bailey JR, Thomas DL, et al Clearance of hepatitis C infection is associated with the early appearance of broad neutralizing antibody responses. Hepatology. 2014;59(6):2140–51. 10.1002/hep.27013 24425349 PMC4043926

[9] Luciani F, Bretana NA, Teutsch S, Amin J, Topp L, Dore GJ, et al A prospective study of hepatitis C incidence in Australian prisoners. Addiction. 2014;109(10):1695–706. 10.1111/add.12643 . 24916002

[10] Ge D, Fellay J, Thompson AJ, Simon JS, Shianna KV, Urban TJ, et al Genetic variation in IL28B predicts hepatitis C treatment-induced viral clearance. Nature. 2009;461(7262):399–401. 10.1038/nature08309 . 19684573

[11] Keck ZY, Xia J, Wang Y, Wang W, Krey T, Prentoe J, et al Human monoclonal antibodies to a novel cluster of conformational epitopes on HCV E2 with resistance to neutralization escape in a genotype 2a isolate. PLoS Pathog. 2012;8(4):e1002653 Epub 2012/04/19. 10.1371/journal.ppat.1002653 PPATHOGENS-D-11-02162 [pii]. 22511875 PMC3325216

[12] Keck Z, Wang W, Wang Y, Lau P, Carlsen TH, Prentoe J, et al Cooperativity in virus neutralization by human monoclonal antibodies to two adjacent regions located at the amino terminus of hepatitis C virus E2 glycoprotein. J Virol. 2013;87(1):37–51. Epub 2012/10/26. 10.1128/JVI.01941-12 23097455 PMC3536422

[13] Keck ZY, Li TK, Xia J, Gal-Tanamy M, Olson O, Li SH, et al Definition of a conserved immunodominant domain on hepatitis C virus E2 glycoprotein by neutralizing human monoclonal antibodies. J Virol. 2008;82(12):6061–6. 10.1128/JVI.02475-07 . 18400849 PMC2395155

[14] Prentoe J, Bukh J. In Vitro Neutralization Assay Using Cultured Hepatitis C Virus. Methods Mol Biol. 2019;1911:433–9. 10.1007/978-1-4939-8976-8_29 . 30593643

[15] Lindenbach BD, Evans MJ, Syder AJ, Wolk B, Tellinghuisen TL, Liu CC, et al Complete replication of hepatitis C virus in cell culture. Science. 2005;309(5734):623–6. 10.1126/science.1114016 . 15947137

[16] Scheel TK, Gottwein JM, Jensen TB, Prentoe JC, Hoegh AM, Alter HJ, et al Development of JFH1-based cell culture systems for hepatitis C virus genotype 4a and evidence for cross-genotype neutralization. Proc Natl Acad Sci U S A. 2008;105(3):997–1002. Epub 2008/01/16. 0711044105 [pii] 10.1073/pnas.0711044105 18195353 PMC2242719

[17] Gottwein JM, Scheel TK, Jensen TB, Lademann JB, Prentoe JC, Knudsen ML, et al Development and characterization of hepatitis C virus genotype 1–7 cell culture systems: role of CD81 and scavenger receptor class B type I and effect of antiviral drugs. Hepatology. 2009;49(2):364–77. 10.1002/hep.22673 . 19148942

[18] Scheel TK, Gottwein JM, Carlsen TH, Li YP, Jensen TB, Spengler U, et al Efficient culture adaptation of hepatitis C virus recombinants with genotype-specific core-NS2 by using previously identified mutations. J Virol. 2011;85(6):2891–906. 10.1128/JVI.01605-10 21177811 PMC3067958

[19] Jensen TB, Gottwein JM, Scheel TK, Hoegh AM, Eugen-Olsen J, Bukh J. Highly efficient JFH1-based cell-culture system for hepatitis C virus genotype 5a: failure of homologous neutralizing-antibody treatment to control infection. J Infect Dis. 2008;198(12):1756–65. 10.1086/593021 . 19032070

[20] Ramirez S, Bukh J. Current status and future development of infectious cell-culture models for the major genotypes of hepatitis C virus: Essential tools in testing of antivirals and emerging vaccine strategies. Antiviral Res. 2018;158:264–87. 10.1016/j.antiviral.2018.07.014 . 30059723

[21] Prentoe J, Jensen TB, Meuleman P, Serre SB, Scheel TK, Leroux-Roels G, et al Hypervariable region 1 differentially impacts viability of hepatitis C virus strains of genotypes 1 to 6 and impairs virus neutralization. J Virol. 2011;85(5):2224–34. 10.1128/JVI.01594-10 21123377 PMC3067759

[22] Pedersen J, Carlsen TH, Prentoe J, Ramirez S, Jensen TB, Forns X, et al Neutralization resistance of hepatitis C virus can be overcome by recombinant human monoclonal antibodies. Hepatology. 2013;58(5):1587–97. 10.1002/hep.26524 23729237 PMC4415732

[23] Prentoe J, Velazquez-Moctezuma R, Foung SK, Law M, Bukh J. Hypervariable region 1 shielding of hepatitis C virus is a main contributor to genotypic differences in neutralization sensitivity. Hepatology. 2016;64(6):1881–92. 10.1002/hep.28705 27351277 PMC5115964

[24] Prentoe J, Bukh J. Hypervariable Region 1 in Envelope Protein 2 of Hepatitis C Virus: A Linchpin in Neutralizing Antibody Evasion and Viral Entry. Front Immunol. 2018;9:2146 10.3389/fimmu.2018.02146 30319614 PMC6170631

[25] Velazquez-Moctezuma R, Law M, Bukh J, Prentoe J. Applying antibody-sensitive hypervariable region 1-deleted hepatitis C virus to the study of escape pathways of neutralizing human monoclonal antibody AR5A. PLoS Pathog. 2017;13(2):e1006214 10.1371/journal.ppat.1006214 28231271 PMC5358973

[26] Ball JK, Tarr AW, McKeating JA. The past, present and future of neutralizing antibodies for hepatitis C virus. Antiviral Res. 2014;105(100):100–11. 10.1016/j.antiviral.2014.02.013 24583033 PMC4034163

[27] Keck ZY, Op De Beeck A, Hadlock KG, Xia J, Li TK, Dubuisson J, et al Hepatitis C virus E2 has three immunogenic domains containing conformational epitopes with distinct properties and biological functions. J Virol. 2004;78(17):9224–32. 10.1128/JVI.78.17.9224-9232.2004 . 15308717 PMC506923

[28] Keck ML, Wrensch F, Pierce BG, Baumert TF, Foung SKH. Mapping Determinants of Virus Neutralization and Viral Escape for Rational Design of a Hepatitis C Virus Vaccine. Front Immunol. 2018;9:1194 10.3389/fimmu.2018.01194 29904384 PMC5991293

[29] Pierce BG, Keck ZY, Lau P, Fauvelle C, Gowthaman R, Baumert TF, et al Global mapping of antibody recognition of the hepatitis C virus E2 glycoprotein: Implications for vaccine design. Proc Natl Acad Sci U S A. 2016 10.1073/pnas.1614942113 27791171 PMC5111724

[30] Gopal R, Jackson K, Tzarum N, Kong L, Ettenger A, Guest J, et al Probing the antigenicity of hepatitis C virus envelope glycoprotein complex by high-throughput mutagenesis. PLoS Pathog. 2017;13(12):e1006735 10.1371/journal.ppat.1006735 29253863 PMC5749897

[31] Velazquez-Moctezuma R, Galli A, Law M, Bukh J, Prentoe J. Hepatitis C Virus Escape Studies of Human Antibody AR3A Reveal a High Barrier to Resistance and Novel Insights on Viral Antibody Evasion Mechanisms. J Virol. 2019;93(4). 10.1128/JVI.01909-18 30487284 PMC6364003

[32] Giang E, Dorner M, Prentoe JC, Dreux M, Evans MJ, Bukh J, et al Human broadly neutralizing antibodies to the envelope glycoprotein complex of hepatitis C virus. Proc Natl Acad Sci U S A. 2012;109(16):6205–10. 10.1073/pnas.1114927109 22492964 PMC3341081

[33] Xiao F, Fofana I, Heydmann L, Barth H, Soulier E, Habersetzer F, et al Hepatitis C virus cell-cell transmission and resistance to direct-acting antiviral agents. PLoS Pathog. 2014;10(5):e1004128 10.1371/journal.ppat.1004128 24830295 PMC4022730

[34] Steinmann E, Doerrbecker J, Friesland M, Riebesehl N, Ginkel C, Hillung J, et al Characterization of hepatitis C virus intra- and intergenotypic chimeras reveals a role of the glycoproteins in virus envelopment. J Virol. 2013;87(24):13297–306. 10.1128/JVI.01708-13 24089562 PMC3838269

[35] Hadlock KG, Lanford RE, Perkins S, Rowe J, Yang Q, Levy S, et al Human monoclonal antibodies that inhibit binding of hepatitis C virus E2 protein to CD81 and recognize conserved conformational epitopes. J Virol. 2000;74(22):10407–16. 10.1128/jvi.74.22.10407-10416.2000 . 11044085 PMC110915

[36] Law M, Maruyama T, Lewis J, Giang E, Tarr AW, Stamataki Z, et al Broadly neutralizing antibodies protect against hepatitis C virus quasispecies challenge. Nat Med. 2008;14(1):25–7. Epub 2007/12/08. nm1698 [pii] 10.1038/nm1698 . 18064037

[37] Merat SJ, Molenkamp R, Wagner K, Koekkoek SM, van de Berg D, Yasuda E, et al Hepatitis C virus Broadly Neutralizing Monoclonal Antibodies Isolated 25 Years after Spontaneous Clearance. PLoS One. 2016;11(10):e0165047 10.1371/journal.pone.0165047 27776169 PMC5077102

[38] Bailey JR, Flyak AI, Cohen VJ, Li H, Wasilewski LN, Snider AE, et al Broadly neutralizing antibodies with few somatic mutations and hepatitis C virus clearance. JCI Insight. 2017;2(9). 10.1172/jci.insight.92872 28469084 PMC5414559

[39] Chan CH, Hadlock KG, Foung SK, Levy S. V(H)1-69 gene is preferentially used by hepatitis C virus-associated B cell lymphomas and by normal B cells responding to the E2 viral antigen. Blood. 2001;97(4):1023–6. . 11159532 10.1182/blood.v97.4.1023

[40] Tzarum N, Giang E, Kong L, He L, Prentoe J, Augestad E, et al Genetic and structural insights into broad neutralization of hepatitis C virus by human VH1-69 antibodies. Sci Adv. 2019;5(1):eaav1882 10.1126/sciadv.aav1882 30613781 PMC6314831

[41] Lang S, Xie J, Zhu X, Wu NC, Lerner RA, Wilson IA. Antibody 27F3 Broadly Targets Influenza A Group 1 and 2 Hemagglutinins through a Further Variation in VH1-69 Antibody Orientation on the HA Stem. Cell Rep. 2017;20(12):2935–43. 10.1016/j.celrep.2017.08.084 28930686 PMC5679313

[42] Kong L, Giang E, Nieusma T, Kadam RU, Cogburn KE, Hua Y, et al Hepatitis C virus E2 envelope glycoprotein core structure. Science. 2013;342(6162):1090–4. Epub 2013/11/30. 342/6162/1090 [pii] 10.1126/science.1243876 . 24288331 PMC3954638

[43] Adolf-Bryfogle J, Xu Q, North B, Lehmann A, Dunbrack RL Jr. PyIgClassify: a database of antibody CDR structural classifications. Nucleic Acids Res. 2015;43(Database issue):D432–8. 10.1093/nar/gku1106 25392411 PMC4383924

[44] Sui J, Hwang WC, Perez S, Wei G, Aird D, Chen LM, et al Structural and functional bases for broad-spectrum neutralization of avian and human influenza A viruses. Nat Struct Mol Biol. 2009;16(3):265–73. Epub 2009/02/24. nsmb.1566 [pii] 10.1038/nsmb.1566 19234466 PMC2692245

[45] Thomson CA, Bryson S, McLean GR, Creagh AL, Pai EF, Schrader JW. Germline V-genes sculpt the binding site of a family of antibodies neutralizing human cytomegalovirus. The EMBO journal. 2008;27(19):2592–602. 10.1038/emboj.2008.179 18772881 PMC2567409

[46] Tarr AW, Owsianka AM, Jayaraj D, Brown RJ, Hickling TP, Irving WL, et al Determination of the human antibody response to the epitope defined by the hepatitis C virus-neutralizing monoclonal antibody AP33. J Gen Virol. 2007;88(Pt 11):2991–3001. Epub 2007/10/20. 88/11/2991 [pii] 10.1099/vir.0.83065-0 . 17947521

[47] Zhang P, Zhong L, Struble EB, Watanabe H, Kachko A, Mihalik K, et al Depletion of interfering antibodies in chronic hepatitis C patients and vaccinated chimpanzees reveals broad cross-genotype neutralizing activity. Proc Natl Acad Sci U S A. 2009;106(18):7537–41. Epub 2009/04/22. 0902749106 [pii] 10.1073/pnas.0902749106 19380744 PMC2670884

[48] Kuiken C, Yusim K, Boykin L, Richardson R. The Los Alamos hepatitis C sequence database. Bioinformatics. 2005;21(3):379–84. 10.1093/bioinformatics/bth485 . 15377502

[49] Scarselli E, Ansuini H, Cerino R, Roccasecca RM, Acali S, Filocamo G, et al The human scavenger receptor class B type I is a novel candidate receptor for the hepatitis C virus. Embo J. 2002;21(19):5017–25. Epub 2002/10/03. 10.1093/emboj/cdf529 12356718 PMC129051

[50] Pileri P, Uematsu Y, Campagnoli S, Galli G, Falugi F, Petracca R, et al Binding of hepatitis C virus to CD81. Science. 1998;282(5390):938–41. . 9794763 10.1126/science.282.5390.938

[51] Douam F, Dao Thi VL, Maurin G, Fresquet J, Mompelat D, Zeisel MB, et al Critical interaction between E1 and E2 glycoproteins determines binding and fusion properties of hepatitis C virus during cell entry. Hepatology. 2014;59(3):776–88. 10.1002/hep.26733 . 24038151

[52] Burioni R, Mancini N, Carletti S, Perotti M, Grieco A, Canducci F, et al Cross-reactive pseudovirus-neutralizing anti-envelope antibodies coexist with antibodies devoid of such activity in persistent hepatitis C virus infection. Virology. 2004;327(2):242–8. 10.1016/j.virol.2004.06.042 . 15351212

[53] Keck ZY, Saha A, Xia J, Wang Y, Lau P, Krey T, et al Mapping a region of hepatitis C virus E2 that is responsible for escape from neutralizing antibodies and a core CD81-binding region that does not tolerate neutralization escape mutations. J Virol. 2011;85(20):10451–63. Epub 2011/08/05. JVI.05259-11 [pii] 10.1128/JVI.05259-11 21813602 PMC3187491

[54] Tarr AW, Lafaye P, Meredith L, Damier-Piolle L, Urbanowicz RA, Meola A, et al An alpaca nanobody inhibits hepatitis C virus entry and cell-to-cell transmission. Hepatology. 2013;58(3):932–9. Epub 2013/04/05. 10.1002/hep.26430 . 23553604

[55] Velazquez-Moctezuma R, Galli A, Law M, Bukh J, Prentoe J. Hepatitis C Virus-Escape Studies for Human Monoclonal Antibody AR4A Reveal Isolate-Specific Resistance and a High Barrier to Resistance. J Infect Dis. 2019;219(1):68–79. 10.1093/infdis/jiy481 . 30102355 PMC6455953

[56] Tillmann HL, Thompson AJ, Patel K, Wiese M, Tenckhoff H, Nischalke HD, et al A polymorphism near IL28B is associated with spontaneous clearance of acute hepatitis C virus and jaundice. Gastroenterology. 2010;139(5):1586–92, 92 e1. 10.1053/j.gastro.2010.07.005 . 20637200

[57] Flyak AI, Ruiz S, Colbert MD, Luong T, Crowe JE Jr., Bailey JR, et al HCV Broadly Neutralizing Antibodies Use a CDRH3 Disulfide Motif to Recognize an E2 Glycoprotein Site that Can Be Targeted for Vaccine Design. Cell Host Microbe. 2018;24(5):703–16 e3. 10.1016/j.chom.2018.10.009 30439340 PMC6258177

[58] Teutsch S, Luciani F, Scheuer N, McCredie L, Hosseiny P, Rawlinson W, et al Incidence of primary hepatitis C infection and risk factors for transmission in an Australian prisoner cohort. BMC Public Health. 2010;10(1):633–41. Epub 2010/10/23. 1471-2458-10-633 [pii] 10.1186/1471-2458-10-633 20964864 PMC2975656

[59] Pham ST, Bull RA, Bennett JM, Rawlinson WD, Dore GJ, Lloyd AR, et al Frequent multiple hepatitis C virus infections among injection drug users in a prison setting. Hepatology. 2010;52(5):1564–72. Epub 2010/11/03. 10.1002/hep.23885 . 21038409

[60] Katoh K, Standley DM. MAFFT multiple sequence alignment software version 7: improvements in performance and usability. Mol Biol Evol. 2013;30(4):772–80. 10.1093/molbev/mst010 23329690 PMC3603318

[61] Bartosch B, Dubuisson J, Cosset FL. Infectious hepatitis C virus pseudo-particles containing functional E1-E2 envelope protein complexes. J Exp Med. 2003;197(5):633–42. 10.1084/jem.20021756 . 12615904 PMC2193821

[62] Carlsen TH, Pedersen J, Prentoe JC, Giang E, Keck ZY, Mikkelsen LS, et al Breadth of neutralization and synergy of clinically relevant human monoclonal antibodies against HCV genotypes 1a, 1b, 2a, 2b, 2c, and 3a. Hepatology. 2014;60(5):1551–62. 10.1002/hep.27298 25043937 PMC4415877

[63] Gottwein JM, Scheel TK, Callendret B, Li YP, Eccleston HB, Engle RE, et al Novel infectious cDNA clones of hepatitis C virus genotype 3a (strain S52) and 4a (strain ED43): genetic analyses and in vivo pathogenesis studies. J Virol. 2010;84(10):5277–93. Epub 2010/03/05. JVI.02667-09 [pii] 10.1128/JVI.02667-09 20200247 PMC2863810

[64] Suzuki R, Shimodaira H. Pvclust: an R package for assessing the uncertainty in hierarchical clustering. Bioinformatics. 2006;22(12):1540–2. 10.1093/bioinformatics/btl117 . 16595560

[65] Paradis E, Claude J, Strimmer K. APE: Analyses of Phylogenetics and Evolution in R language. Bioinformatics. 2004;20(2):289–90. 10.1093/bioinformatics/btg412 . 14734327

[66] Brochet X, Lefranc MP, Giudicelli V. IMGT/V-QUEST: the highly customized and integrated system for IG and TR standardized V-J and V-D-J sequence analysis. Nucleic Acids Res. 2008;36(Web Server issue):W503–8. 10.1093/nar/gkn316 18503082 PMC2447746

[67] Beaumont T, Merat SJL, Schinkel CJ, Molenkamp R, inventors; AIMM Therapeutics B.V., assignee. Hepatitis C virus specific antibody. US patent application 2017/0313764 A1. November 2, 2017.

