# Supplementary figures and images for: Broadly neutralizing antibodies from an individual that naturally cleared multiple hepatitis C virus infections uncover molecular determinants for E2 targeting and vaccine design
Source: PLoS Pathog. 2019 May 17;15(5):e1007772. doi: 10.1371/journal.ppat.1007772 (PMC6542541; doi:10.1371/journal.ppat.1007772)

**S1 Figure**

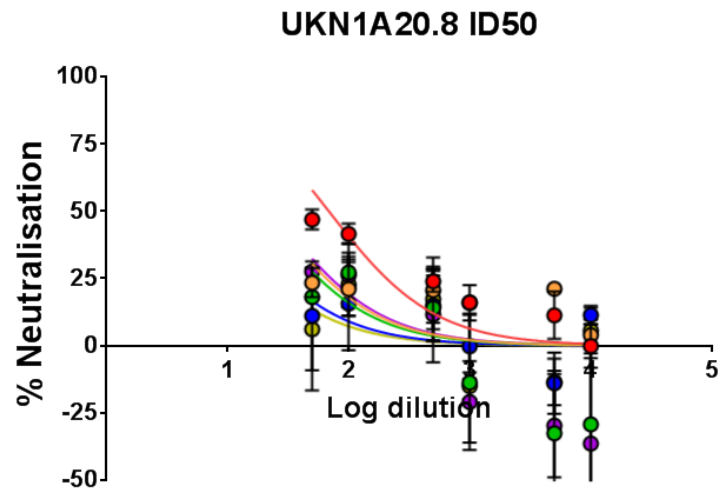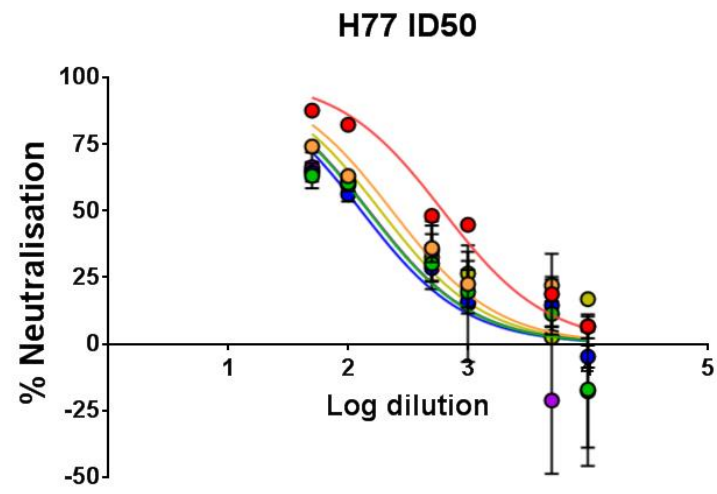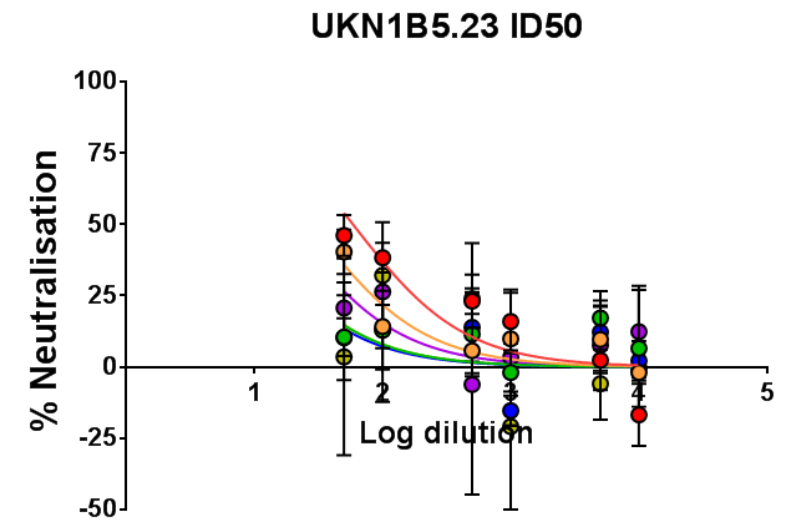

- Week 76
- Week 122
- Week 123
- Week 135
- Week 150
- Week 182

Supplement: S1 Fig — The % neutralization (y-axis) was plotted against the log10 serum dilution (x-axis) for all defined time points listed in the figure legends. A non-linear regression analysis (log inhibitor vs normalized response) was performed in GraphPad PRISM v7.02 to calculate the estimated inhibitory concentration required to neutralize 50% of the virus (IC50). The % neutralization was calculated by comparing the RLU of healthy serum to the RLU of the tested serum (1-[RLU test serum]/[RLU healthy serum]). (PDF) [file ppat.1007772.s001.pdf]

## S2 Figure

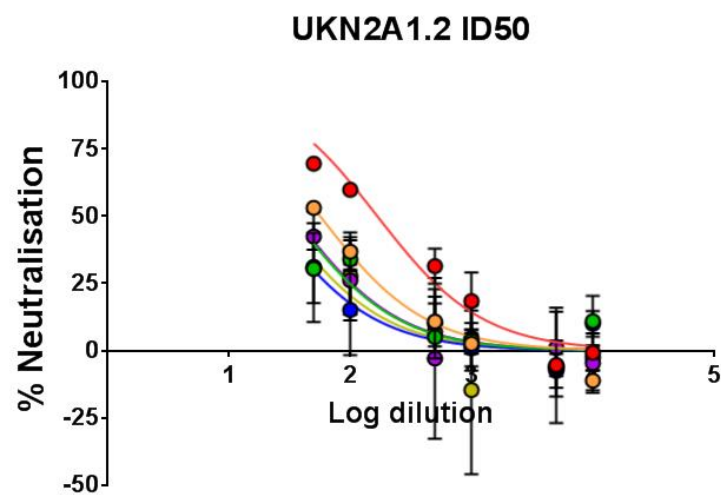

- Week 76
- Week 122
- Week 123
- Week 135
- Week 150
- Week 182

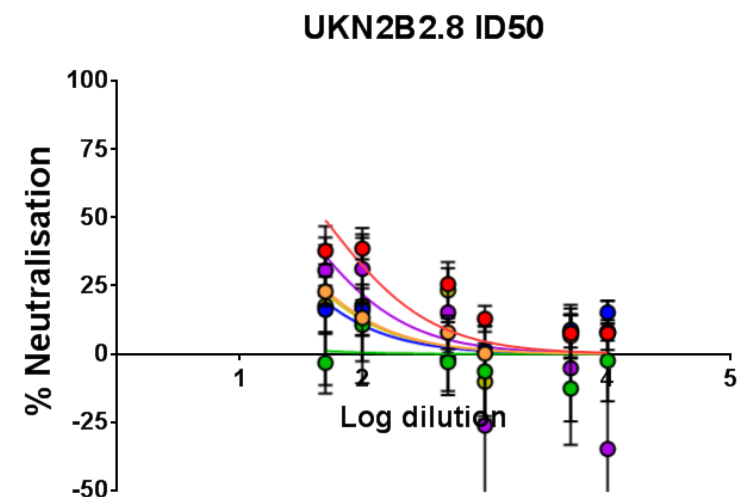

Supplement: S2 Fig — The % neutralization (y-axis) was plotted against the log10 serum dilution (x-axis) for all defined time points listed in the figure legends. A non-linear regression analysis (log inhibitor vs normalized response) was performed in GraphPad PRISM v7.02 to calculate the estimated inhibitory concentration required to neutralize 50% of the virus (IC50). The % neutralization was calculated by comparing the RLU of healthy serum to the RLU of the tested serum (1-[RLU test serum]/[RLU healthy serum]). (PDF) [file ppat.1007772.s002.pdf]

## S3 Figure

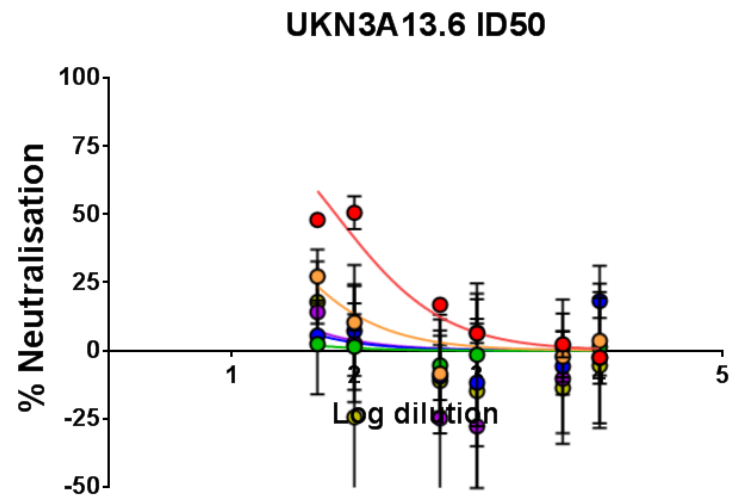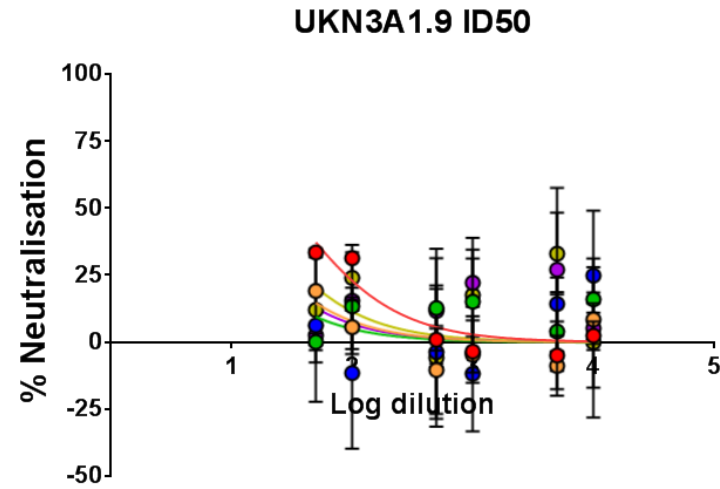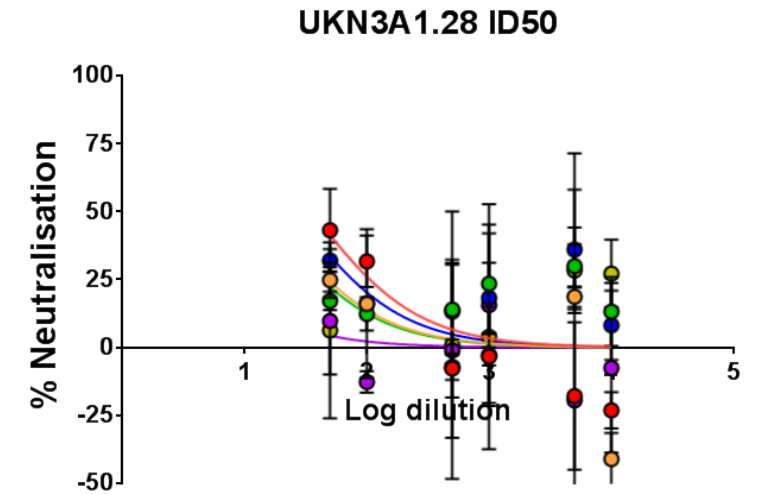

- Week 76
- Week 122
- Week 123
- Week 135
- Week 150
- Week 182

Supplement: S3 Fig — The % neutralization (y-axis) was plotted against the log10 serum dilution (x-axis) for all defined time points listed in the figure legends. A non-linear regression analysis (log inhibitor vs normalized response) was performed in GraphPad PRISM v7.02 to calculate the estimated inhibitory concentration required to neutralize 50% of the virus (IC50). The % neutralization was calculated by comparing the RLU of healthy serum to the RLU of the tested serum (1-[RLU test serum]/[RLU healthy serum]). (PDF) [file ppat.1007772.s003.pdf]

S4 Figure

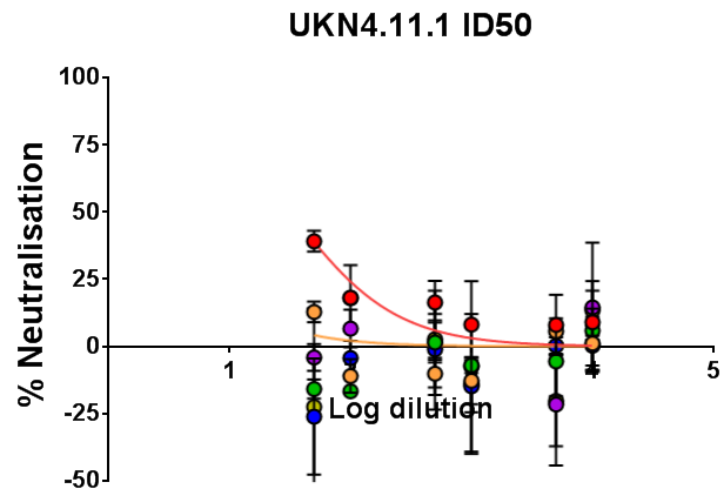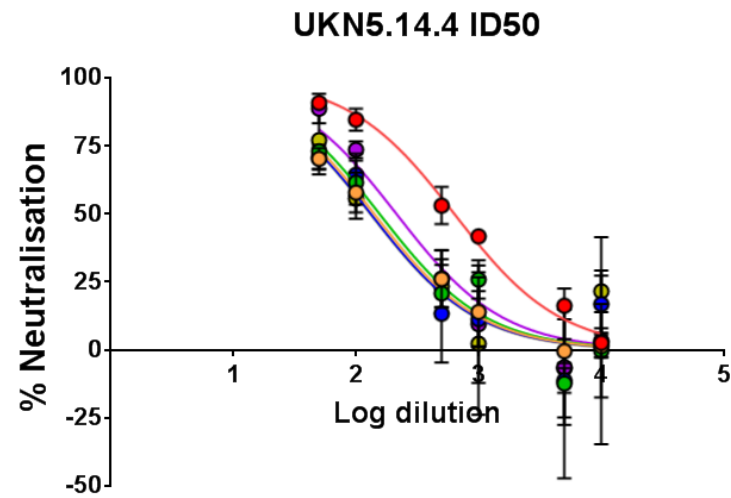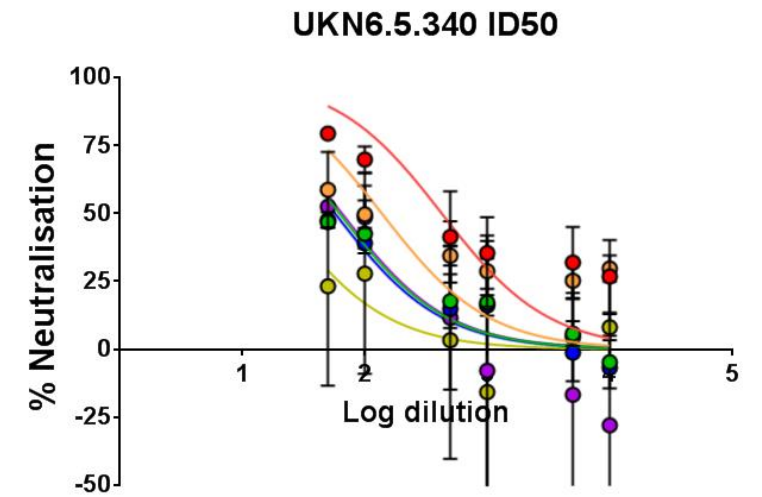

- Week 76
- Week 122
- Week 123
- Week 135
- Week 150
- Week 182

Supplement: S4 Fig — The % neutralization (y-axis) was plotted against the log10 serum dilution (x-axis) for all defined time points listed in the figure legends. A non-linear regression analysis (log inhibitor vs normalized response) was performed in GraphPad PRISM v7.02 to calculate the estimated inhibitory concentration required to neutralize 50% of the virus (IC50). The % neutralization was calculated by comparing the RLU of healthy serum to the RLU of the tested serum (1-[RLU test serum]/[RLU healthy serum]). (PDF) [file ppat.1007772.s004.pdf]

S5 Figure

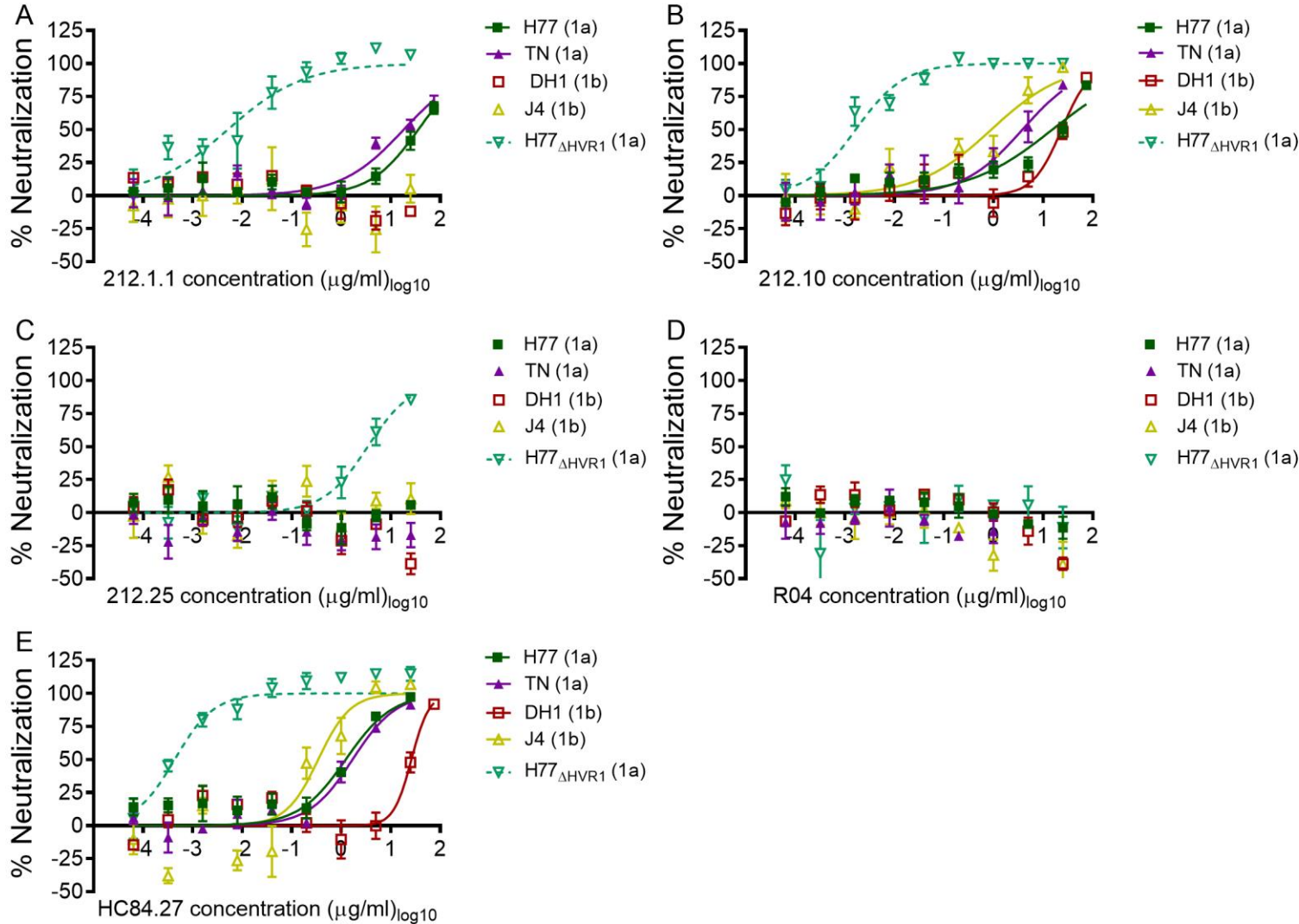

Supplement: S5 Fig — Virus stocks of the indicated genotypes 1a and 1b HCV Core-NS2 recombinants were subjected to dose-response FFU reduction neutralization assays using dilution series of the antibodies (A) 212.1.1, (B) 212.10, (C) 212.25, (D) R04, or (E) HC84.27 in quadruplicates with 8 wells of virus only. Following a total of 48 hours infection the cells were immuno-stained and the number of FFUs per well were counted as described in Materials and Methods. Error bars represent standard error of the mean of four replicates normalized to 8 replicates of virus only. The data was analyzed using four-parameter curve-fitting to obtain a sigmoidal dose-response curve, permitting the interpolation of an IC50 value (Graphpad PRISM 7.02). J4 and H77ΔHVR1 gave fewer than 20 FFUs/well in virus only wells against all antibodies. (PDF) [file ppat.1007772.s005.pdf]

**S6 Figure**

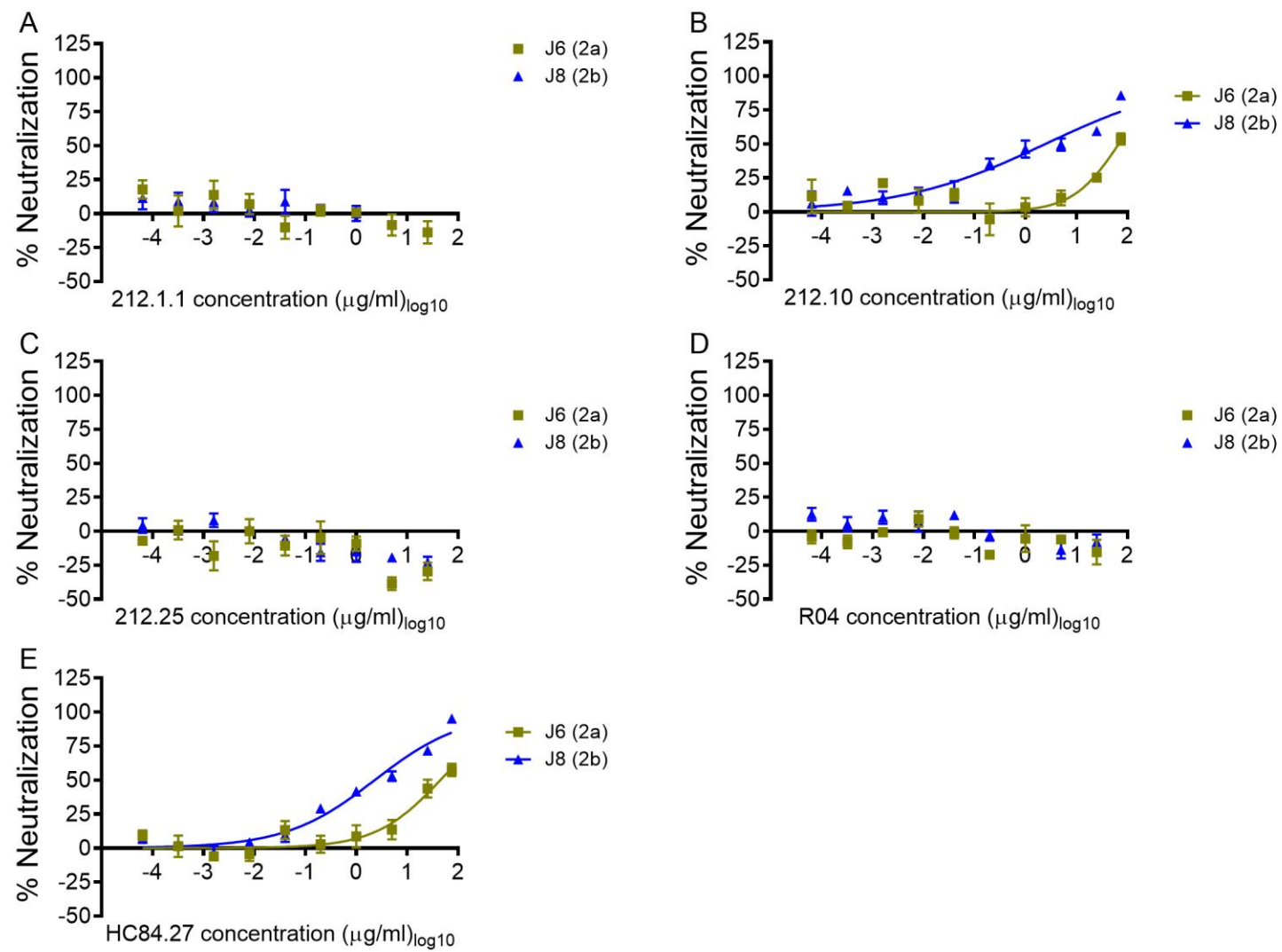

Supplement: S6 Fig — Virus stocks of the indicated genotypes 2a and 2b HCV Core-NS2 recombinants were subjected to dose-response FFU reduction neutralization assays using dilution series of the antibodies (A) 212.1.1, (B) 212.10, (C) 212.25, (D) R04, or (E) HC84.27 in quadruplicates with 8 wells of virus only. Following a total of 48 hours infection the cells were immuno-stained and the number of FFUs per well were counted as described in Materials and Methods. Error bars represent standard error of the mean of four replicates normalized to 8 replicates of virus only. The data was analyzed using four-parameter curve-fitting to obtain a sigmoidal dose-response curve, permitting the interpolation of an IC50 value (Graphpad PRISM 7.02). (PDF) [file ppat.1007772.s006.pdf]

**S7 Figure**

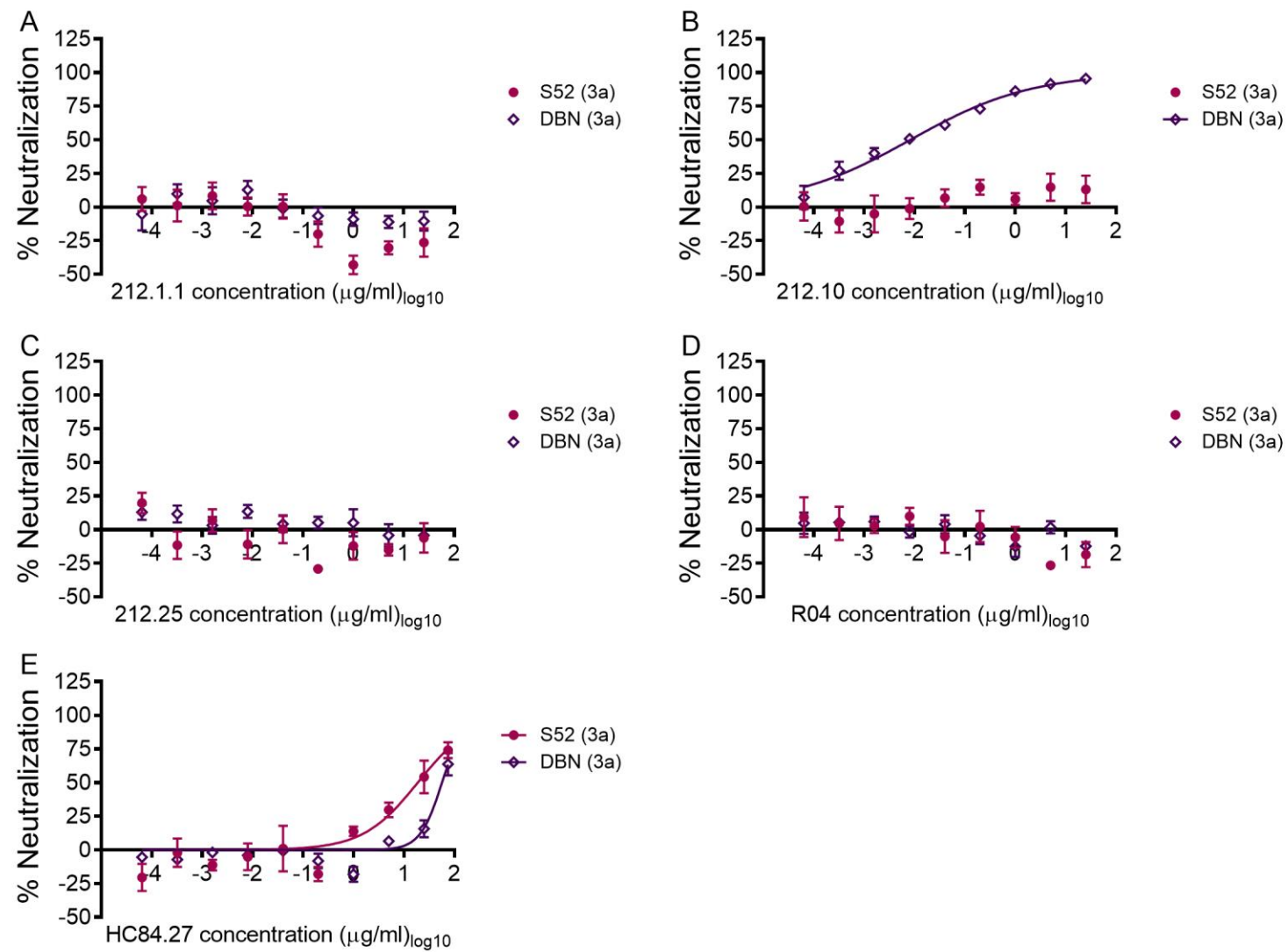

Supplement: S7 Fig — Virus stocks of the indicated genotype 3a HCV Core-NS2 recombinants were subjected to dose-response FFU reduction neutralization assays using dilution series of the antibodies (A) 212.1.1, (B) 212.10, (C) 212.25, (D) R04, or (E) HC84.27 in quadruplicates with 8 wells of virus only. Following a total of 48 hours infection the cells were immuno-stained and the number of FFUs per well were counted as described in Materials and Methods. Error bars represent standard error of the mean of four replicates normalized to 8 replicates of virus only. The data was analyzed using four-parameter curve-fitting to obtain a sigmoidal dose-response curve, permitting the interpolation of an IC50 value (Graphpad PRISM 7.02). (PDF) [file ppat.1007772.s007.pdf]

**S8 Figure**

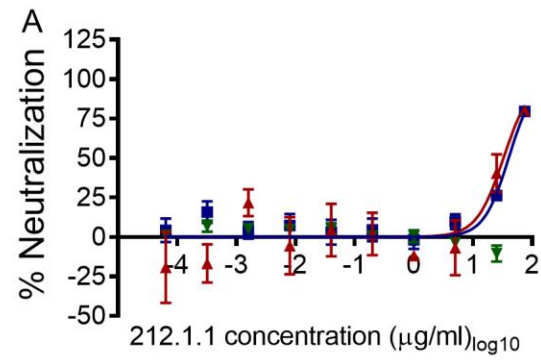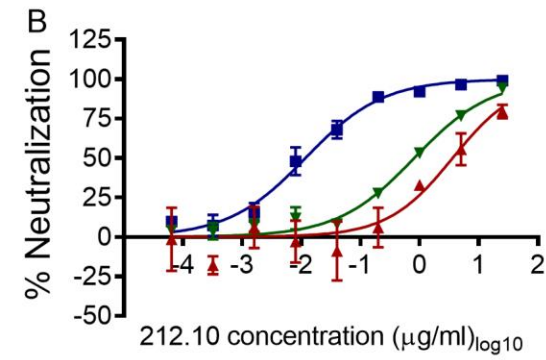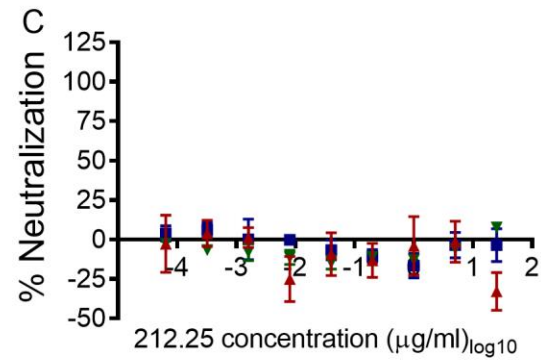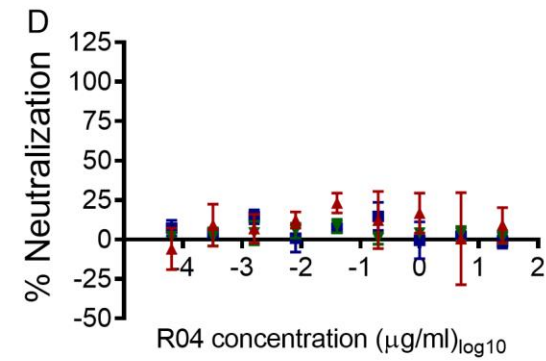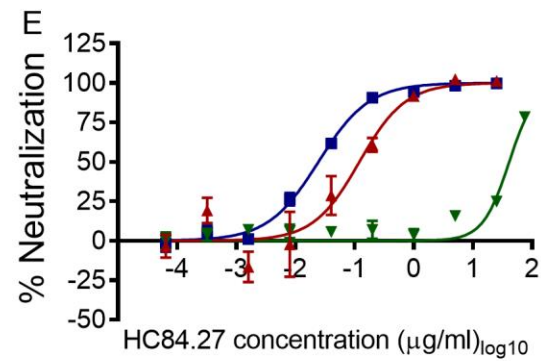

Supplement: S8 Fig — Virus stocks of the indicated genotypes 4a, 5a, or 6a HCV Core-NS2 recombinants were subjected to dose-response FFU reduction neutralization assays using dilution series of the antibodies (A) 212.1.1, (B) 212.10, (C) 212.25, (D) R04, or (E) HC84.27 in quadruplicates with 8 wells of virus only. Following a total of 48 hours infection the cells were immuno-stained and the number of FFUs per well were counted as described in Materials and Methods. Error bars represent standard error of the mean of four replicates normalized to 8 replicates of virus only. The data was analyzed using four-parameter curve-fitting to obtain a sigmoidal dose-response curve, permitting the interpolation of an IC50 value (Graphpad PRISM 7.02). ED43 gave fewer than 20 FFUs/well in virus only wells against 212.10 and 212.25 and SA13 gave more than 200 (but no more than 210) FFUs/well in virus only wells against 212.1.1, R04, and HC84.27. (PDF) [file ppat.1007772.s008.pdf]

S9 Figure

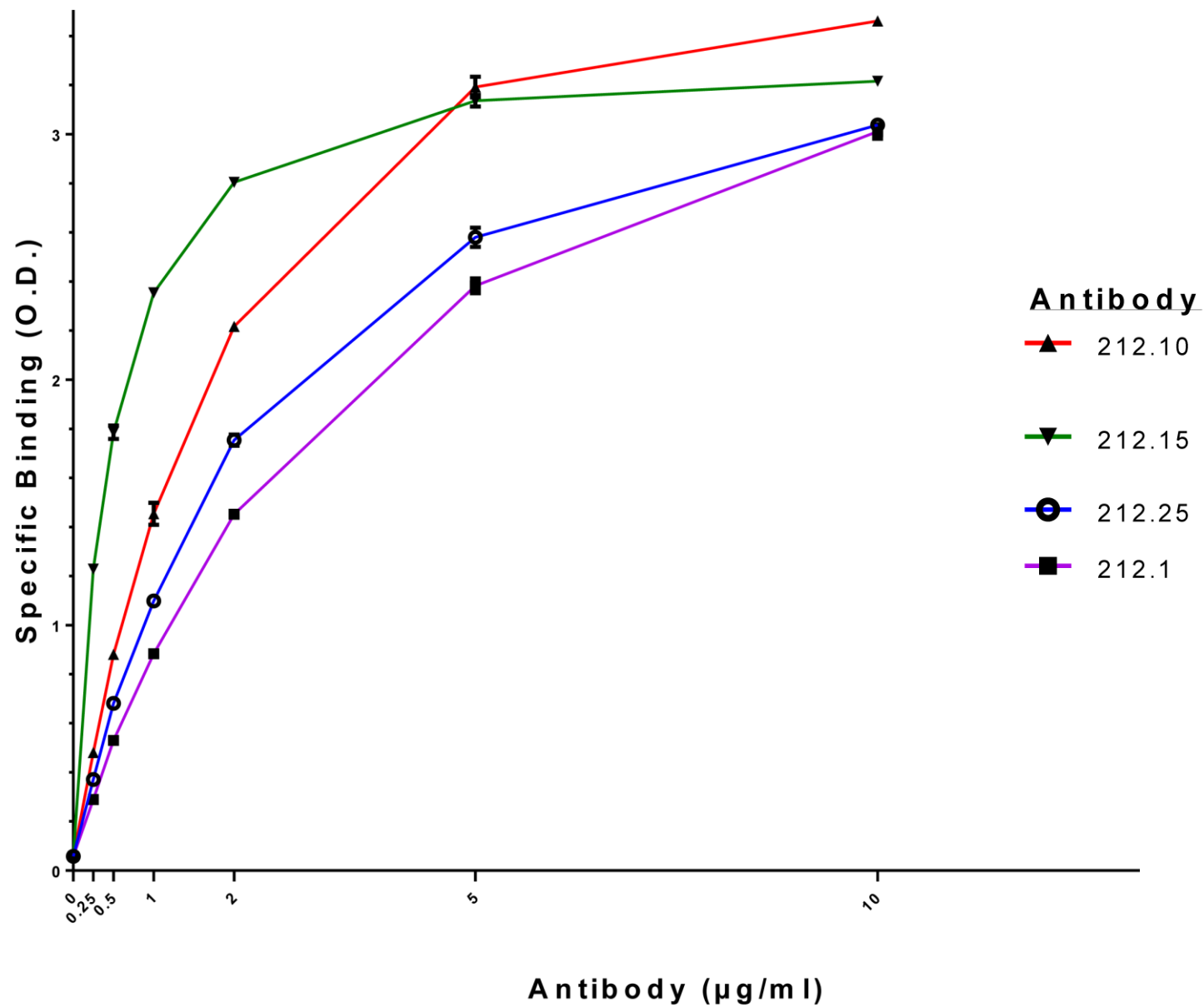

Supplement: S9 Fig — Recombinant H77C 1a E1E2 lysates were captured by pre-coated GNA wells. After washing and blocking, bound proteins were incubated with each indicated 212 HMAb at 0.005–2 μg/ml (x-axis) for 30 minutes. After washing, bound antibodies were detected as described in Materials and Methods. The y-axis shows the mean optical density values for triplicate wells, the mean of two experiments ± SD. (PDF) [file ppat.1007772.s009.pdf]

**S10 Figure**

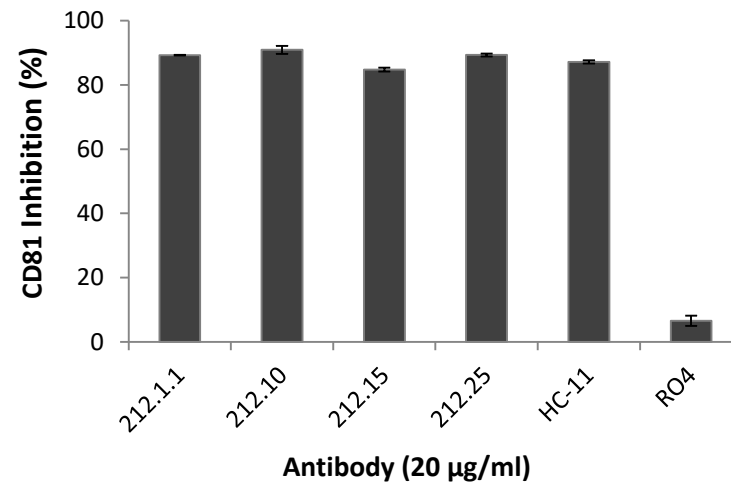

Supplement: S10 Fig — H77C 1a recombinant E1E2 lysate containing 1 μg/ml E2 was incubated with each test HMAb at 10 μg/ml. The antibody-antigen complex was then added onto CD81-LEL-precoated wells. Detection of E2 bound to CD81-LEL was measured with biotinylated CBH-4D [13, 35–38]. HC-11 was used as a positive control and R04 as a negative control (a HMAb to HCMV). Inhibition of binding is expressed as percent inhibition (y-axis). Experiments were performed twice in triplicate. Error bars indicate one standard deviation from the mean. (PDF) [file ppat.1007772.s010.pdf]

## S11 Figure

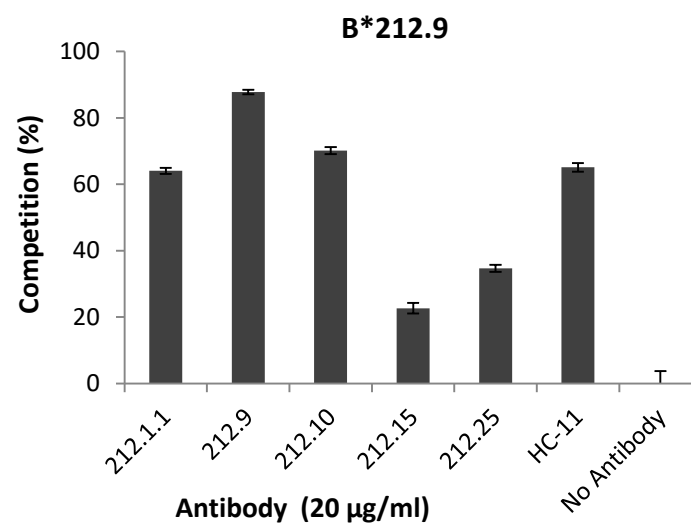

Supplement: S11 Fig — Recombinant autologous 1b E1E2 lysates were captured by pre-coated GNA wells. After washing and blocking, bound proteins were incubated with each indicated 212 blocking antibody at 20 μg/ml (x-axis), a control antigenic domain B HMAb, HC-11, and a no antibody control for 30 minutes. After washing, labeled 212.9 HMAb at 2 μg/ml was added. Bound 212. 9 HMAb was detected as described in Materials and Methods. The y-axis shows the percent competition by each blocking antibody, the mean of two experiments ±SD that were performed in triplicates. (PDF) [file ppat.1007772.s011.pdf]

**S12 Figure**

**A**

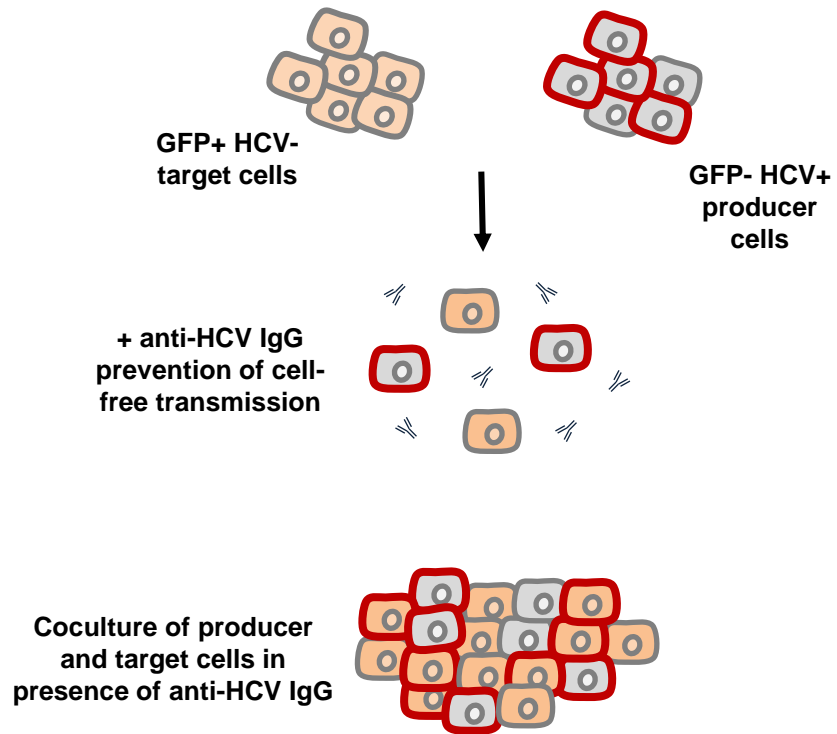

**B**

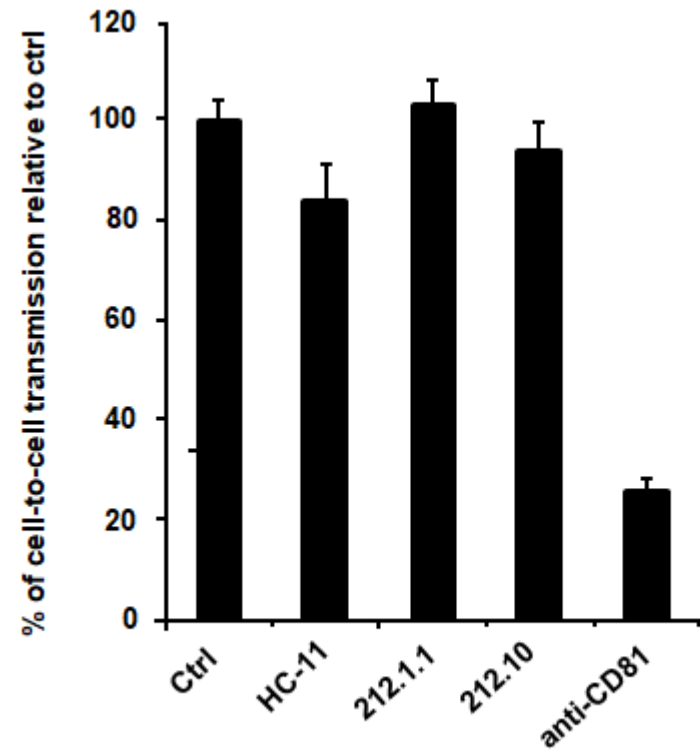

Supplement: S12 Fig — (A) Approach of HCV cell-cell transmission experiments. HCV Huh7.5.1 producer cells cultured with naïve Huh7.5-GFP target cells were incubated with control or anti-HCV HMAbs (100 μg/ml) in the presence of anti-HCV IgG (50 μg/ml) to block cell-free transmission similar as described [33]. Cell-to-cell transmission was determined by quantification of HCV+ GFP+ target cells using immunostaining and flow cytometry. (B) HCV cell-cell transmission indicated as percentage of HCV-infected Huh7.5-GFP target cells is shown as histogram. Means +/- SD from two independent experiments performed in duplicate are shown. Incubation of cells with anti-CD81 MAb served as positive control. (PDF) [file ppat.1007772.s012.pdf]
