# Supplementary material for: Broadly neutralizing antibodies from an individual that naturally cleared multiple hepatitis C virus infections uncover molecular determinants for E2 targeting and vaccine design
Source: PLoS Pathog. 2019 May 17;15(5):e1007772. doi: 10.1371/journal.ppat.1007772 (PMC6542541; doi:10.1371/journal.ppat.1007772)
Supplement: S1 Table — HCV RNA was detected as described in Materials and Methods. (PDF) [file ppat.1007772.s013.pdf]

S1 Table

Chronology of multiple HCV infections

Timepoint*	**21**	**76**	**122**	**123**	**135**	**150**	**161**	**182**
HCV Infection	**1b RNA**	--	**1a RNA**	--	--	--	--	--
HCV Ab	+	+	+	+	+	+	+	+

Time Point	**203**	**236**	**256**	**277**	**278**	**291**	**303**	**320**
HCV Infection	--	--	--	**3a RNA**	+	+	+	--
HCV Ab	+	+	+	+	+	+	+	+

** Weeks after the initial evaluation*
