# Supplementary material for: Broadly neutralizing antibodies from an individual that naturally cleared multiple hepatitis C virus infections uncover molecular determinants for E2 targeting and vaccine design
Source: PLoS Pathog. 2019 May 17;15(5):e1007772. doi: 10.1371/journal.ppat.1007772 (PMC6542541; doi:10.1371/journal.ppat.1007772)
Supplement: S2 Table — A standard ELISA against of native E1E2 glycoproteins was performed as described in Materials and Methods. (PDF) [file ppat.1007772.s014.pdf]

S2 Table

Antibody binding to different HCV genotypes and autologous isolates

	H77c 1a	2a	212 1b	1b	2b	3a	4a	5a	6a
212.1.1	+++	--	++	--	--	+	--	--	--
212.9	--	+++	++++	++++	+++	--	+	++++	++++
212.10	++++	+++	++	++++	++	++	+++	++++	+++
212.15	++++	+++	++	++	+++	--	++	+	+++
212.25	++++	++	++	++++	+	+	+	+	++
CBH-5	++++	+++	+++	+++	++++	++++	++++	++++	++++
