# Supplementary material for: Broadly neutralizing antibodies from an individual that naturally cleared multiple hepatitis C virus infections uncover molecular determinants for E2 targeting and vaccine design
Source: PLoS Pathog. 2019 May 17;15(5):e1007772. doi: 10.1371/journal.ppat.1007772 (PMC6542541; doi:10.1371/journal.ppat.1007772)
Supplement: S3 Table — This study was performed as described in Materials and Methods. (PDF) [file ppat.1007772.s015.pdf]

S3 Table

Competition of 212 antibody binding to E2

		Biotinylated Antibody (2 μg/ml)			
		HC-11	CBH-7	HC84.27	HC33.1
Blocking HMAb (20 μg/ml)	212.1.1	62*	65	60	38
	212.9	66	42	63	33
	212.10	65	65	64	35
	212.15	46	83	54	1
	212.25	46	83	52	1

* Percent inhibition
